# Bridging Gaps in Malaysian Acne Vulgaris Guidelines: Advisory Statements on Trifarotene for Facial and Truncal Acne

**DOI:** 10.1111/jocd.70625

**Published:** 2025-12-28

**Authors:** Azura Mohd Affandi, Peter Wee Beng Ch'ng, Benji Tze Yuen Teoh, Evelyn Wen Yee Yap, Chin Chwen Ch'ng, Seow Hoong Foo, Kang Nien How, Zhenli Kwan, Jyh Jong Tang, Wooi Chiang Tan, Khen Kon, Wei Thian Poh

**Affiliations:** ^1^ Department of Dermatology Hospital Kuala Lumpur Kuala Lumpur Ministry of Health Malaysia; ^2^ Peter Ch'ng Clinic Kuala Lumpur Malaysia; ^3^ Central Dermatology Kuala Lumpur Malaysia; ^4^ Mahkota Medical Centre Melaka Malaysia; ^5^ Department of Dermatology Subang Jaya Medical Centre Subang Jaya Malaysia; ^6^ Gleneagles Hospital Johor Iskandar Puteri Malaysia; ^7^ Dermatology Unit, Faculty of Medicine and Health Sciences Universiti Putra Malaysia Serdang Malaysia; ^8^ Division of Dermatology, Department of Medicine Universiti Malaya Kuala Lumpur Malaysia; ^9^ Department of Dermatology Hospital Raja Permaisuri Bainun Ipoh Ministry of Health Malaysia; ^10^ Department of Dermatology Hospital Pulau Pinang George Town Ministry of Health Malaysia; ^11^ Galderma Sydney Australia; ^12^ Galderma Petaling Jaya Malaysia

**Keywords:** acne vulgaris, evidence‐based practice, retinoids, trifarotene

## Abstract

**Background:**

Trifarotene, a fourth‐generation topical retinoid, offers a novel therapeutic option for facial and truncal acne. However, its role was only briefly addressed in Malaysia's 2022 Clinical Practice Guidelines (CPGs) due to its unavailability at the time. This paper aims to provide expert advisory statements on the integration of trifarotene into acne management in Malaysia, addressing evidence gaps in guideline coverage, particularly for truncal acne and acne‐induced sequelae.

**Methods:**

A literature review was conducted to synthesize evidence on trifarotene's efficacy, safety, and clinical positioning. An advisory panel of 10 Malaysian dermatologists reviewed the evidence and provided consensus through structured surveys and discussion meetings. Three illustrative case studies were included to demonstrate the real‐world application of trifarotene.

**Results:**

Ten advisory statements were finalized, addressing trifarotene's role in acne algorithms, patient selection, dosing strategies, combination therapy, and management of acne sequelae. Evidence supports its use for both facial and truncal acne, with additional benefits in acne‐induced hyperpigmentation and atrophic scarring. The panel recommended practical approaches to optimize tolerability and adherence, such as the CTMP (Cleanse‐Treat‐Moisturize‐Photoprotect) regimen and incremental application strategies. Real‐world cases demonstrated trifarotene's utility in diverse clinical settings.

**Conclusion:**

These advisory statements provide clinical guidance on the use of trifarotene in Malaysia and support its integration into routine acne management. As current CPGs may remain unchanged in the near term, these recommendations aim to inform evidence‐based practice while bridging gaps in local dermatologic care.

## 1 Introduction

Acne vulgaris is one of the most common dermatological conditions worldwide, with a clinical spectrum ranging from mild non‐inflammatory lesions to severe inflammatory presentations [1, 2]. A study based on an analysis of data from the Global Disease Burden Study 2021 reported an estimated prevalence rate of 9790.5 (95% uncertainty interval 8420.9–11 287.2) per 100 000 population in 2021 among adolescents and young adults [3]. Beyond its physical manifestations, acne can have a profound impact on a person's quality of life (QoL), self‐esteem, and psychosocial well‐being [4, 5, 6]. Consequently, the management of acne extends beyond dermatologists in clinical settings, involving general practitioners, aesthetic medicine practitioners, and other allied healthcare providers across a variety of practice settings [7, 8].

In Malaysia, efforts to standardize acne management culminated in the publication of updated local clinical practice guidelines (CPGs) in 2022 [7]. The current guideline emphasizes core principles of acne care, including topical therapies (such as retinoids and benzoyl peroxide [BPO]) and the appropriate use of systemic agents for more severe disease. In recent years, trifarotene (AKLIEF), a fourth‐generation topical retinoid, has garnered increasing recognition for its demonstrated efficacy and safety in the treatment of both facial and truncal acne [9]. Given that most existing acne treatment studies focused on facial areas, trifarotene's proven efficacy on the trunk areas directly addressed an evidence gap, offering a much‐needed therapeutic option for managing truncal acne that is often challenging to manage yet clinically important to address. However, the role of trifarotene in acne management was not extensively addressed in the CPGs, as it was not available in the Malaysian market at the time of guideline development.

Key characteristics of trifarotene include its targeted mechanism of action and minimal systemic absorption, rendering it suitable for large treatment areas such as the trunk [10]. Additionally, emerging evidence highlights its potential benefits in accelerating the improvement of acne‐induced hyperpigmentation and atrophic scars—outcomes of particular relevance to patient quality of life and satisfaction [11, 12]. In light of these advancements, there is a growing need for clinical guidance on the use of trifarotene for the management of both truncal and facial acne.

Given that the current Malaysian Acne Clinical Practice Guidelines are expected to remain in use for an extended period, this paper seeks to provide a timely advisory statement on the role of trifarotene in clinical practice based on the latest published evidence on trifarotene alongside expert insights from a panel of experienced dermatologists in Malaysia. Specifically, this paper aims to: (1) clarify how trifarotene can be integrated into existing acne management algorithms for both facial and truncal areas, (2) outline practical considerations for patient selection, dosing regimens, and combination therapy, particularly in populations with higher risk of acne‐induced hyperpigmentation or scarring, and (3) highlight strategies to improve adherence and long‐term treatment outcomes.

## 2 Materials and Methods

### 2.1 Literature Review

A targeted literature review was conducted from May to August 2024 to identify and synthesize evidence on trifarotene in acne management, including its efficacy, safety, dosing, and positioning relative to other therapies. This review was guided by a pre‐specified framework (see Appendix S1), which delineated key areas of interest such as facial and truncal acne, hyperpigmentation and scarring, combination therapy, and special populations.

Literature searches were conducted in PubMed, Cochrane Library, and Google Scholar. The search employed both keywords and subject headings, encompassing terms such as “acne,” “trifarotene,” “CD5789,” and using Boolean operators (and, or) to combine them. An initial screening of titles and abstracts was performed to determine relevance based on the pre‐specified framework, after which potentially eligible articles were retrieved in full text for detailed evaluation.

Studies or reviews focusing on trifarotene's clinical use in acne (covering both facial and truncal involvement) were considered if they reported outcomes pertaining to lesion reduction, safety profiles, patient satisfaction, and/or acne‐induced hyperpigmentation or scarring. Only English‐language publications were included. Articles were excluded if they did not focus on acne vulgaris.

All relevant literature was assessed and synthesized per the framework's thematic areas (e.g., efficacy in truncal acne, combination therapy strategies). Evidence tables were constructed to summarize study designs, populations, interventions, and main outcomes.

### 2.2 Development of the Advisory Statements

Upon completion of the literature review, the extracted evidence was systematically mapped to each topic in the framework (e.g., “Trifarotene's Role in Acne Treatment,” “Management of Side Effects,” “Use in Special Populations”). Preliminary advisory statements were then crafted to reflect the consolidated findings.

Ten experienced dermatologists from the private and public health facilities, as well as academic institutions, in Malaysia were invited and agreed to form an advisory panel. A draft of the advisory statements was circulated to the members of the advisory panel for their initial feedback. An online survey was administered in which panel members were asked to rate their level of agreement (1 = strongly disagree; 5 = strongly agree) to each advisory statement. The survey also included open‐ended questions for any qualitative feedback, allowing panelists to suggest additional evidence, clarify ambiguities, or refine statements.

Following the survey, a face‐to‐face meeting was conducted with the advisory panel to review the compiled feedback and agreement statistics (i.e., distribution of responses). Each statement was revisited considering the panel's quantitative and qualitative inputs. Further revisions were proposed and discussed during this meeting until a consensus was reached.

Revised statements incorporating feedback from the meeting were then circulated for a final round of review. Any remaining points of disagreement were resolved through one‐on‐one consultations and subsequent updates, followed by a re‐circulation to all panel members. A statement was considered final when all (100%) participants indicated full agreement (i.e., no further modifications needed).

### 2.3 Case Studies

Three illustrative case studies were retrospectively identified to represent different clinical scenarios in which trifarotene was utilized (e.g., varying severity of acne, presence of truncal involvement, or presence of acne sequalae). For each case, relevant patient demographics, clinical history, therapeutic regimen (including trifarotene application details and adjunctive treatments), and outcomes were systematically documented. Follow‐up assessments were conducted to evaluate improvements in acne lesions, tolerability concerns (e.g., local irritation), and patient‐reported satisfaction. All patient data were de‐identified to ensure confidentiality, and written informed consent was obtained for the publication of clinical details and images. These case studies were synthesized to highlight practical lessons learned and to show the diverse contexts in which trifarotene can be effectively integrated into acne management.

## 3 Results

Table 1 presents these statements, organized in alignment with the key themes in acne management and the stated goals of this advisory. All 10 advisory statements were finalized with unanimous agreement among panel members. These statements form a cohesive framework for integrating trifarotene into routine acne care. They collectively address the role of trifarotene as a valuable addition to Malaysia's acne armamentarium and how it can be integrated effectively to our acne management for treating inflammatory lesions and non‐inflammatory lesions, minimizing irritation, and improving long‐term outcomes.

**TABLE 1:** Evidence‐based advisory statements on trifarotene for acne management.

Advisory statement	Key evidence/rationale	Practical clinical notes
**1. Integrate trifarotene into existing acne management algorithms** *“Trifarotene, a fourth‐generation topical retinoid is recommended for both facial and/or truncal acne, positioned alongside established first‐line agents (*e.g., *other retinoids, benzoyl peroxide). Its favorable safety profile and low systemic absorption make it particularly suitable for large treatment areas.”*	**Novel mechanism**: Trifarotene selectively targets RARγ receptors, prevalent in the skin, modulating gene expression of known retinoid pathways (e.g., reducing inflammatory mediators such as CXCL13, SPP1, MMP12, MMP13) and modulate novel pathways involved in cell adhesion, skin hydration and proteolysis [13, 14, 15]. **Pharmacokinetics**: Studies indicate that trifarotene is rapidly metabolized by liver enzymes, with a hepatic half‐life of 5 min. This swift degradation reduces the potential for systemic accumulation and associated side effects, even when applied to extensive skin areas [13]. **Clinical trials**: PERFECT 1 & 2 [16], DUAL [17], START [11], LEAP [12] & Blume‐Peytavi et al. [18] show significant reductions in both inflammatory and non‐inflammatory lesions, plus improved scarring and acne‐induced hyperpigmentation outcomes. **Comparison gap**: Direct comparative trials between trifarotene and other retinoids have not been reported. Indirect comparisons with the third‐generation retinoid tazarotene (facial) showed no significant differences in reductions in lesion counts or treatment success rates after 12 weeks of treatment [4]. However, pre‐clinical findings suggest better potency may be expected for trifarotene [13].	**Algorithm positioning**: Can be used as monotherapy for mild‐to‐moderate acne or combined with systemic agents for more severe cases [16, 17]. **Long‐term potential**: Data support safe and sustained efficacy for extended durations (≥ 12 weeks) up to 52 weeks [11, 12, 16, 17, 18]. **Drug interaction**: No clinically significant drug–drug interactions were identified. Exposure to trifarotene did not affect the performance of oral contraceptives (levonorgestrel 0.15 mg/ethinyl estradiol 0.03 mg) [10, 19]. **Guideline gap**: Current Malaysian guideline does not specifically address trifarotene [7]. This statement provides interim clinical guidance.
**2. Patient selection criteria** *“Trifarotene is suitable for adolescents (≥ 12 years old, per Malaysia regulatory approval) and adults with mild‐to‐moderate facial and/or truncal acne, including those prone to hyperpigmentation or atrophic scarring. It is not recommended as monotherapy for nodulocystic acne that may require systemic interventions.”*	**Efficacy across acne severity**: Effective to treat mild‐to‐moderate facial and truncal acne with both inflammatory and non‐inflammatory lesions [16]. Combination with systemic agents has also shown efficacy to treat moderate‐to‐severe acne [17]. **Hyperpigmentation/scarring**: Particularly beneficial for patients at risk of atrophic scars [11] or acne‐induced hyperpigmentation [12]; faster depigmenting activity reported vs. older retinoids in preclinical study [13]. **Exclusions**: Nodulocystic acne, a severe form of acne characterized by deep, inflamed cysts, typically requires systemic therapies, as topical agents are generally insufficient for managing this condition [1, 2].	**Clinical assessment**: Evaluate lesion type (inflammatory vs. non‐inflammatory), severity (CASS: grade 2 (mild), grade 3 (moderate), grade 4 (severe), and scarring risk before prescribing. **Real‐world insight**: Panelists highlighted truncal involvement and scarring as key indications for trifarotene use, particularly in cases where older topical retinoids are less well‐studied.
**3. Dosage and administration** *“Apply a thin layer of trifarotene cream at night on cleansed, dry skin, extending beyond visible lesions. A minimum duration of 12 weeks is recommended to achieve optimal improvements. Incremental introduction schedules (*e.g., *alternate‐day or brief‐contact) can reduce initial irritation.”*	**Consistency and long‐term use**: Clinical trials, including the PERFECT 1 and 2 studies, demonstrate that trifarotene requires continuous application for 12 weeks to achieve significant lesion reduction and improvement in both facial and truncal acne [16]. The gradual onset of action is consistent with the pharmacodynamics of topical retinoids, which promote cell turnover and dermal remodeling over time [13]. **Addressing skin barrier needs**: Studies show early weeks [1, 2, 3, 4] may bring mild‐to‐moderate dryness, erythema, or stinging, peaking on the face by week 1 and on the trunk by weeks 2–4 [16, 18]. **Real‐world insights**: Limited data from small case series suggest that trifarotene is associated with high patient satisfaction, particularly regarding ease of application and improvements in facial and truncal acne [5]. This evidence is based on small‐scale research, and larger, controlled trials do not consistently measure, or report detailed satisfaction scores. More robust, long‐term data are needed to confirm these findings.	**Application to clean, dry skin**: At least 20–30 min after cleansing. **Practical dosing**: 1 pump for the entire face; 2 pumps for the upper trunk; additional pumps if treating mid/lower back. To minimize anticipated adverse effects such as peeling, erythema, and irritation, clinicians may consider initiating treatment with a reduced dose, based on clinical judgment. **Truncal acne application challenge**: As truncal acne affects areas that are not visible by patient, assistance by caregiver or tool to ease application.
**4. Combination therapy (other topical agents & systemic agents for acne)** *“For moderate‐to‐severe acne, combining trifarotene with oral antibiotics (*e.g., *doxycycline) can enhance efficacy. Trifarotene can also be combined with other topical acne treatments (*e.g., *benzoyl peroxide, azelaic acid).”*	**Benzoyl peroxide (BPO)**: A study observed chemical instability of retinoids like tretinoin when used in combination with BPO, underscoring the importance of considering the chemical compatibility and stability of products used in combination therapies [20]. However, specific studies on the chemical stability of trifarotene when combined with BPO are not available. **Oral antibiotics**: Clinical evidence supports synergy in severe acne. In the DUAL study, trifarotene plus oral doxycycline was demonstrated to be efficacious in reducing lesion counts compared to vehicle plus placebo for patients with severe facial acne [17].	**Combined approach**: **BPO**: Following the common recommendation to apply BPO and retinoids, like tretinoin, at separate times (e.g., BPO in the morning, tretinoin at night) [21], a similar approach could be considered for trifarotene. **Systemic antibiotics**: Start trifarotene concurrently with systemic antibiotics in moderate‐to‐severe cases; re‐evaluate after 8–12 weeks for down‐titration. **Caution**: Monitor dryness or irritation when multiple topicals are used (e.g., BPO, topical antibiotics). Educate patients on using each product at different times of day, if possible, to minimize overlap and irritation. **Use with oral isotretinoin**: Panelists acknowledged varying clinical practices regarding the concomitant use of oral isotretinoin and topical retinoids. The bridging method—tapering or discontinuing oral isotretinoin as acne improves, followed by gradual introduction of topical retinoids—is more widely adopted. Small‐scale studies suggest topical retinoids (e.g., tazarotene, tretinoin) reduce relapse rates in this setting [22, 23, 24]. Although no large‐scale or trifarotene‐specific data are available, panelists agreed that this approach could be considered for trifarotene.
**5. Role of trifarotene in the management of acne sequelae** *“Trifarotene is recommended as a topical therapy for managing acne sequelae in patients with acne‐induced scarring and acne‐induced hyperpigmentation. Combining trifarotene with adjunctive modalities may enhance outcomes, although further validation is needed.”*	**Dual action in active acne and sequelae**: The START study [11] demonstrated trifarotene's efficacy in reducing active acne lesions (inflammatory and non‐inflammatory) and improving preexisting atrophic scars, with significant results by week 2, continuing through week 24. Participants had moderate‐to‐severe active acne and mild‐to‐moderate scars. Similarly, the LEAP study [12] showed significant reductions in acne‐induced hyperpigmentation overall disease severity (ODS) score by week 12, and significant reduction in post‐acne vulgaris hyperpigmentation index (PAHPI) score at week 24. LEAP participants had moderate acne with acne‐induced hyperpigmentation associated with active lesions. Both studies support trifarotene's use in managing acne sequelae as a consequence of active acne; no evidence supports its standalone use for scars or acne‐induced hyperpigmentation. **Mechanism of action**: Trifarotene selectively targets RARγ receptors, modulating collagen remodeling, epidermal turnover, and inflammation reduction, effectively addressing active acne lesions and sequelae like atrophic scars and acne‐induced hyperpigmentation [12, 25, 26]. **Combination modalities**: Evidence from systematic reviews suggests that combining treatments, such as chemical peels and fillers, has the potential to enhance outcomes [27, 28, 29]. However, no studies investigating the combination of trifarotene with these modalities have been reported, and this topic is scarcely addressed in clinical guidelines, with the exception of limited data on hyaluronic acid fillers. Preliminary evidence from small case series indicates that the sequential use of hyaluronic acid fillers with trifarotene may enhance outcomes for scars, although further research is needed [25].	**Potential benefit in acne‐induced erythema**: Trifarotene has shown potential benefits in reducing acne‐induced erythema, which is more noticeable in lighter skin phototypes, as observed in the LEAP study [12]. However, since erythema reduction was not a predefined endpoint, findings were reported descriptively. Further validation through dedicated clinical trials assessing its effect on acne‐induced erythema reduction is needed. **Scar management**: For persistent atrophic scars, layering retinoid therapy with filler injections spaced ~1 month apart may be an option [25].
**6. Management of side effects** *“Initial erythema, dryness, or stinging commonly occurs in the first 1–4 weeks, with severity peaking sooner on the face than the trunk. These effects are generally mild and mitigated by gentle cleanser, moisturizer and photoprotection, incremental dosing, and patient education on expected skin changes.”*	**Local tolerability**: Local adverse effects consistent with known pattern of topical retinoid dermatitis; trunk applications often better tolerated than facial use [11, 16, 18]. **Common AEs**: Clinical studies (PERFECT 1 and 2 trials) [16] show that trifarotene is well‐tolerated, with application site irritation (7.5%), pruritus (2.4%), and sunburn (2.6%) as the most common adverse reactions [30]. These effects are typically mild to moderate, transient, and rarely lead to discontinuation (1.9% and 1.2% in the PERFECT 1 and 2 trials respectively) [16]. A 52‐week study further confirmed its safety, with 12.6% experiencing treatment‐emergent adverse events (TEAEs) but none of which were classified as serious. Irritation peaked in week 1 for the face and weeks 2–4 for the trunk, decreasing over time. Severe signs and symptoms of local tolerability were low (2.2%–7.1% face; 2.5%–5.4% trunk) [18]. **Transient nature**: Symptoms typically improve with continued use [18]. **Photoprotection**: Topical retinoids increase the risk of phototoxicity, and UV radiation can exacerbate acne flare‐ups [31]. It is recommended to use sunscreen with SPF 30 or higher that provides both UVA and UVB protection and to wear protective clothing [32]. **Optimum use of cleansers and moisturizers**: Improve tolerability and adherence to topical treatments [32].	**Acne flare‐ups after retinoid initiation**: Flare‐ups or transient worsening of acne have been reported after starting topical retinoids, such as tretinoin, although these occurrences are not commonly documented in published literature [33, 34, 35]. Patient reassurance is crucial to manage expectations and prevent premature discontinuation. **Cleanse‐treat‐moisturize‐photoprotect (CTMP) routine** [32] (The routine may vary based on clinical assessment): **Gentle cleanser** (non‐comedogenic): Use twice daily to remove dirt and sebum. **Trifarotene treatment** **Moisturizer** (non‐comedogenic, preferably alcohol free, non‐greasy, water‐based): Moisturize twice daily to prevent dryness from retinoids **Photoprotection**: SPF 30 or higher, UVA + UVB protection; protective clothes. **Frequency adjustments**: Temporarily reduce application frequency (e.g., every other night) if patients experience significant irritation. **Special populations**: Lower trans‐epidermal water loss (TEWL) in some Asian skin types can heighten irritation to chemical stimuli [36]—advise incremental or brief‐contact approaches. **Patient alerts**: Avoid applying to broken skin or sunburned areas [37].
**7. Use in special populations** *“Trifarotene is supported by safety data in adolescents (≥ 12 years). Contraindications include pregnancy and lactation.”*	**Adolescent & adult populations**: Trials included a wide age range, showing favorable efficacy and tolerability in patients ≥ 9 years and adults [16, 18]. Approved in some regions from age 9 (US) [30] or 12 (Europe, Malaysia [19]). **Darker skin types**: Darker skin types are more prone to acne‐induced hyperpigmentation [12]. Clinical studies involving patients with darker skin types have shown reductions in hyperpigmentation and scarring [11, 12], but subgroup‐specific analysis and large‐scale real‐world data are limited. **Pregnancy/lactation**: Minimal systemic absorption [10], but safety evidence is insufficient. Clinical consensus recommends discontinuation if pregnancy is confirmed. Caution is advised when considering the use of topical retinoids during breastfeeding [19]. **Exclusions**: Limited data in pregnancy/lactation (The manufacturer makes no recommendation regarding use during pregnancy).	**Use in 9 to 11‐year‐olds**: Pharmacokinetic (PK) data indicate similar drug accumulation patterns in both pediatric and adult patients [10]. Additionally, efficacy and safety trials for trifarotene included participants aged 9–11 years, although this group had a small sample size and no subgroup data were reported [16, 18]. The efficacy and safety of trifarotene for this age group require further validation in future studies. **Tailored approach**: Use incremental application methods in those prone to hyperpigmentation and irritation (e.g., deeper skin phototypes, Asians with lower TEWL). **Clinical monitoring**: Encourage early follow‐up to assess response and side effects, especially in adolescents starting retinoid therapy for the first time.
**8. Patient education and adherence** *“Comprehensive, patient‐centric education on proper application techniques, realistic timelines (≥ 8–12 weeks), and CTMP (Cleanse‐Treat‐Moisturize‐Protect) skincare regimen significantly improves adherence. Reduced application, brief‐contact or layering methods can be introduced for those with sensitive or easily irritated skin.”*	**Adherence imperative**: A recent study shows > 90% of trifarotene users find moisturizers and gentle cleansers help reduce dryness and irritation, while improve compliance [12]. Experience from PERFECT 1 & 2 trials also suggested that the integration of routine skincare, including non‐comedogenic moisturizers and gentle cleansers, helps mitigate local tolerability issues, and promote sustained adherence in most patients [16]. **Psychosocial factors**: Patient satisfaction is an essential treatment goal, as it plays a critical role in improving adherence. This is influenced by multiple factors, including satisfaction with acne improvement and appearance [8]. Providing patients with information about how acne treatments function and the time required to see results, combined with clear guidance on skincare routines, helps manage patient expectations and improve outcomes [38]. **Support materials**: An earlier study found that patients on acne treatment who received supplementary educational materials, including videos and information cards exhibited significantly greater adherence and satisfaction [39]. A local study also reported that medical education and counseling improved treatment adherence to topical treatment and disease severity among patients with acne vulgaris [6]. These studies align with a panel of dermatologists' opinions. In an expert consensus, they emphasized the importance of improving patient education on skin condition, training support staff for patient counseling, and offering physician training opportunities. These efforts aim to enhance patients' understanding of their treatment, ensure proper product selection, and enhance compliance and outcomes [32].	**Gradual onboarding and incremental application**: In practice, many dermatologists initiate with 2–3 nights per week, especially for younger or sensitive‐skin patients. Common clinical strategies include as below (tailored according to clinical assessment): Week 1: 2 times per week at night Week 2: 3 times per week at night Week 3: 4 times per week at night Following week onwards: every night **Brief‐contact**: Brief‐contact therapy (≤ 30 min before rinsing) can be employed to minimize irritation for people with sensitive skin [26]. **Skincare regimen**: Incorporating a comprehensive skincare regimen—consisting of a gentle cleanser, acne treatment, moisturizer, and photoprotection (CTMP) (See Statement 6: Management of Side Effects) — helps optimize acne management and enhance treatment adherence. Inadequate or inappropriate skincare can exacerbate acne vulgaris, increase irritation from treatments, and disrupt sebum production [31, 32]. **Moisturizer layering**: The use of moisturizer is recommended and patients are advised to follow the “open sandwich” moisturisation regimen —applying moisturizer either before or after trifarotene, but not both [19, 40]. For normal skin, apply moisturizer either before or after trifarotene cream; for oily skin, apply moisturizer after trifarotene cream. Advisable to wait ≥ 1 h after moisturizer to apply trifarotene (or vice versa) to minimize product dilution [4]. **Spot initiation approach**: Apply trifarotene to a small area to assess tolerability before gradually extending to broader field application. **Follow‐up & engagement**: Shorter follow‐up visits initially to address side effects, reaffirm correct usage, and adjust frequencies; consider harnessing social media or phone apps for reminders. **Support materials**: Multilingual or visual aids (online platforms, printed leaflets) will likely help align patient expectations under the diverse ethnic and cultural environment in Malaysia.
**9. Maintenance therapy** *“Trifarotene may be considered for maintenance therapy in patients with acne that has achieved clinical clearance. However, data explicitly supporting trifarotene's role as maintenance treatment beyond 52 weeks is not available.”*	A 24‐week split face vehicle‐controlled trial assessed trifarotene's effect on atrophic acne scars in patients with moderate‐to‐severe facial acne [11]: **Efficacy**: The trial reported a statistically significantly greater reduction in the total atrophic scar count at 24 weeks in the trifarotene‐treated area compared to vehicle‐treated area, with differences noted as early as Week 2. Both SGA and IGA success rates were significantly higher in trifarotene‐treated side at Week 24. **Safety**: The incidence of TEAEs was 5.8% in the trifarotene‐treated area and 2.5% in the vehicle‐treated area. All events were mild to moderate in severity. A 52‐week study [18] in patients with moderate facial and truncal acne evaluated the long‐term safety and efficacy of trifarotene: **Efficacy**: Progressive improvement in both facial and truncal acne over time, although the study design required treatment discontinuation upon lesion clearance, limiting data on post‐clearance maintenance therapy. **Safety and tolerability**: Trifarotene was generally well tolerated, TEAEs being mild to moderate. Only 3.5% of TEAEs led to treatment discontinuation, with the majority (2.9%) occurring in the first 3 months, during which most TEAEs occurred. Local irritation peaked early—Week 1 for the face and Weeks 2–4 for the trunk—before decreasing with continued use. Tolerability generally improved over time, supporting its suitability for extended treatment.	**Trifarotene as maintenance therapy**: Although data on trifarotene as maintenance therapy post‐acne clearance are not explicitly available, panelists agreed that it is suitable for maintenance therapy to sustain clearance and prevent relapse. This is consistent with the panelists' practice of using other retinoids as maintenance treatment, with duration guided by clinical discretion and patient response.
**10. Addressing unmet needs & future directions** *“Trifarotene extends effective topical therapy to truncal acne, improving atrophic scars and hyperpigmentation in diverse populations. Further comparative research and real‐world data are needed to refine guidelines, especially for combination regimens (*e.g., *with benzoyl peroxide) and special populations.”*	**Truncal acne**: Truncal acne is often underdiagnosed [41], with 48%–52% of patients with facial acne also experiencing truncal acne [9]. Data on management of truncal acne are limited [42], with limited development until the PERFECT 1 and PERFECT 2 studies. This area is often neglected in existing guidelines [11, 16, 18, 36]. However, recent clinical programs (PERFECT 1 & 2 [16], START [11], Blume‐Peytavi et al. [18]) addressed trunk involvement, and guidelines specifically for truncal acne are emerging [42]. **Fast improvement in atrophic scars & hyperpigmentation**: Clinical data indicate early changes in atrophic scars (as soon as week 2) and hyperpigmented lesions with trifarotene compared to vehicle cream [11, 12]. This evidence suggests that trifarotene offers a rapid onset of action in improving atrophic scars and hyperpigmented lesions, potentially exceeding the efficacy of older retinoids in these parameters. **Research gap**: Limited direct comparisons with other topical retinoids (adapalene, tretinoin), role of trifarotene in acne‐induced erythema and stable acne scar, unclear synergy with BPO and energy‐based devices, as well as insufficient data on pregnant and lactating women.	**Guideline evolution**: Future revision of the Malaysian guidelines could incorporate dedicated recommendations for truncal acne. **Future studies**: Real‐world evidence (RWE) on large‐scale patient populations—particularly those with comorbidities or prior retinoid intolerance—provide clearer insights on trifarotene's role. **Clinical implementation**: Panel members concur that bridging gaps in data and providing education for non‐dermatologist clinicians will enhance patient access to treatment and improve outcomes.

For a detailed breakdown of the underlying survey data, literature references, and extended panel discussions, please refer to the Appendix S1.

### 3.1 Case Studies

Three illustrative case studies were selected to demonstrate the practical application of trifarotene in differing clinical scenarios. Each case highlights specific aspects of treatment and outcomes, underscoring the versatility and challenges of trifarotene use in real‐world settings.

#### 3.1.1 Case 1: Monotherapy for Moderate‐to‐Severe Facial and Truncal Acne

An 11‐year‐old Chinese male patient presented with moderate‐to‐severe acne vulgaris (CASS grade 3) affecting both the face and trunk. The facial lesions were predominantly located on the forehead and bilateral cheeks, while truncal acne was observed on the upper back, particularly in the area below the neck. Baseline images depicted multiple inflammatory papules and comedones across the affected areas.

**FIGURE 1:**
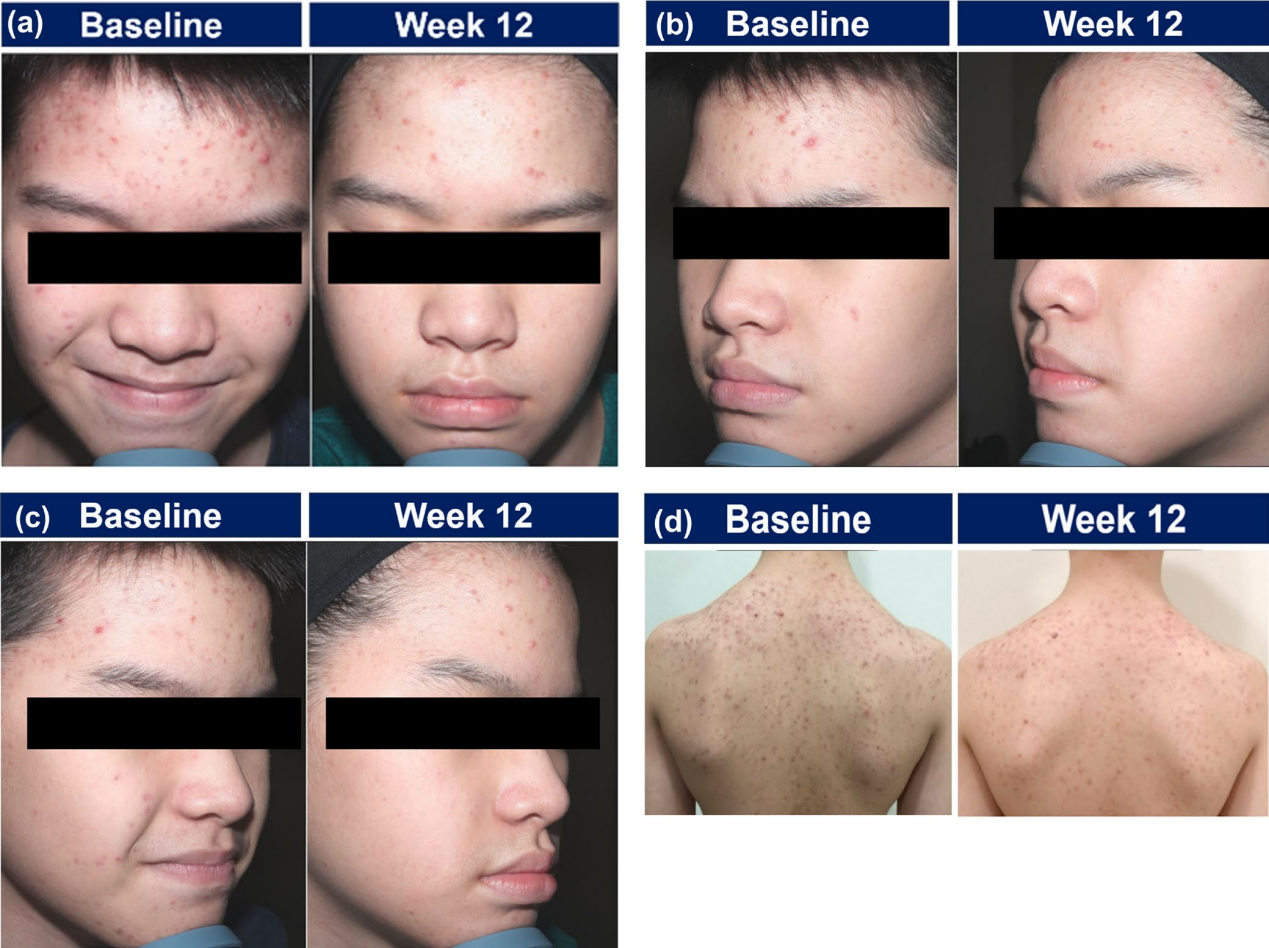
Clinical improvement in acne lesions following trifarotene therapy on face (a–c) and trunk (d) for Case 1.

The patient had no history of prior acne treatments and was initiated on topical trifarotene monotherapy alongside a CTMP regimen. The treatment was applied to both the face and trunk, with instructions to apply a moisturizer before trifarotene to mitigate irritation. Counseling was provided on adherence and proper application techniques. During the first week, the patient reported mild dryness and irritation, prompting a temporary reduction in treatment frequency to every other night for 2 weeks. After 2 weeks, the patient resumed nightly application as tolerated. The patient was compliant with the treatment regimen. Follow‐up at 12 weeks demonstrated notable clinical improvement in both facial and truncal acne (CASS grade 1). Inflammatory lesions were significantly reduced, with a clearer skin appearance observed across treated areas. The patient expressed satisfaction with the treatment outcome, and no serious adverse effects were reported (Figure 1). The patient was continued on topical trifarotene monotherapy for maintenance.

This case demonstrates the use of trifarotene monotherapy in managing moderate‐to‐severe facial and truncal acne. It highlights how complementing treatment with CTMP regimen can support tolerability, enabling consistent use of trifarotene and contributing to clinical improvement.

#### 3.1.2 Case 2: Combination Topical Therapy for Facial Acne

A 23‐year‐old Malay female presented with mild facial acne (CASS grade 2), characterized by multiple inflammatory acne lesions, comedones, acne‐induced erythema and acne‐induced hyperpigmentation. She had no prior acne treatment but had been using over‐the‐counter skincare products. The patient was prescribed a topical‐only regimen consisting of trifarotene and azelaic acid 20% cream. Trifarotene was selected over tretinoin due to the patient's prior experience of irritation with tretinoin. Trifarotene was applied nightly to the entire face, followed by a moisturizer, while azelaic acid was used as a spot treatment twice daily. She was initially recommended to start with alternate‐day application of trifarotene, but the patient opted for daily use and tolerated it well. She was counseled on potential irritation and advised to skip a day of treatment if symptoms occurred, which she followed when necessary. Sunscreen was also recommended.

**FIGURE 2:**
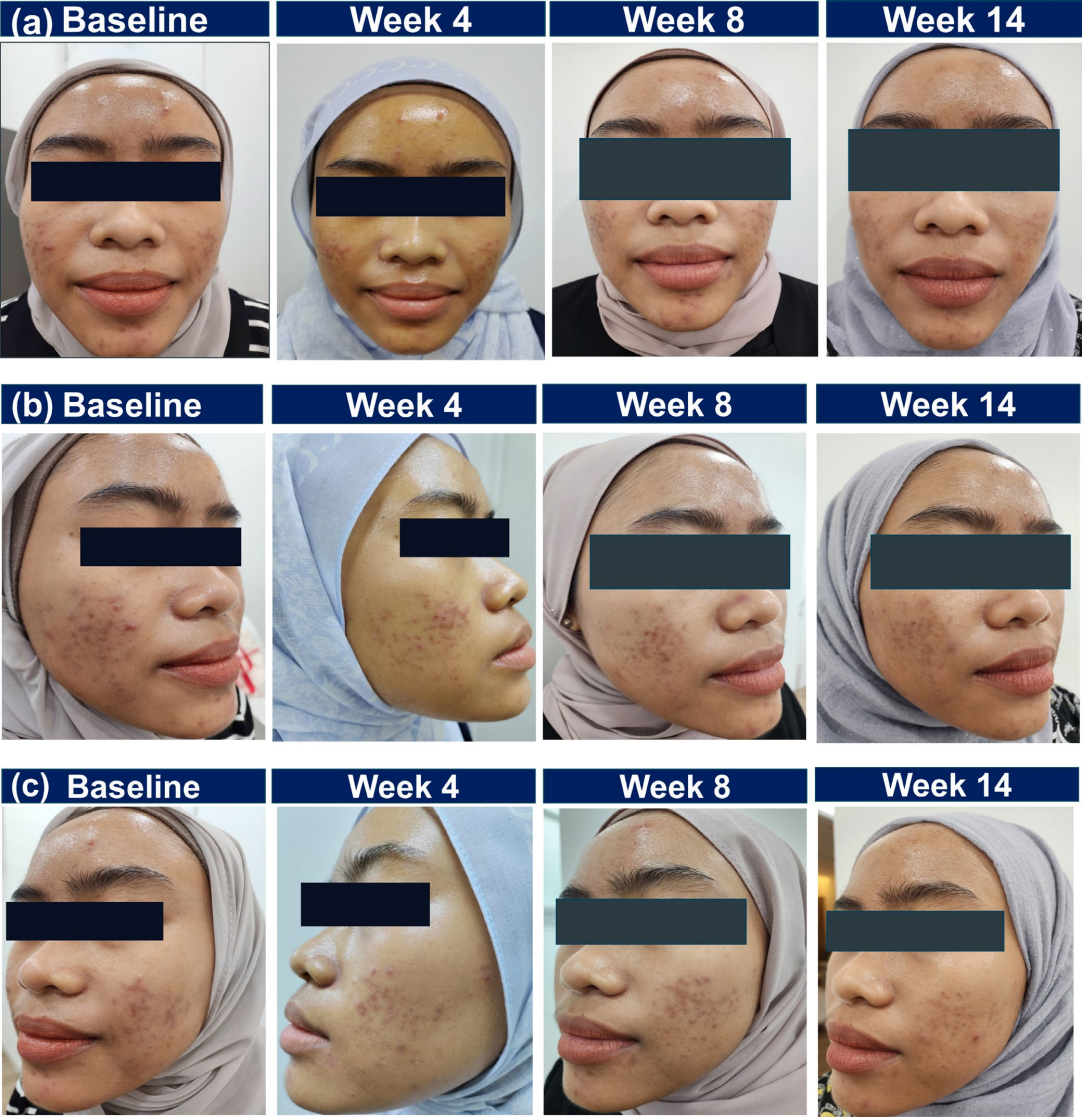
Clinical improvement in acne lesions, acne‐induced erythema, and acne‐induced hyperpigmentation following trifarotene and azelaic acid combination therapy on face (a–c) for Case 2.

By Week 4, notable improvement was observed, including a reduction in acne lesion count and visible fading of erythema and hyperpigmentation. At the Week 8 follow‐up, the patient demonstrated sustained improvement, characterized by a reduction in comedones and the presence of only one remaining active lesion (Figure 2). By Week 14, continued progress was observed, with CASS scores as follows: chin and forehead scored 1; right cheek scored between 0 and 1; left cheek scored between 0 and 1. The patient demonstrated high compliance with treatment and reported satisfaction with the outcomes. As of the last follow‐up, she remained on daily trifarotene therapy followed by moisturizer, as maintenance therapy.

This case demonstrates the potential of combining trifarotene with another topical agent in managing mild‐to‐moderate facial acne with acne‐induced erythema and hyperpigmentation. It highlighted how patient‐led adjustments to application frequency can enhance treatment tolerability and compliance while maintaining treatment efficacy in both active acne lesions and acne‐induced sequelae.

#### 3.1.3 Case 3: Combination of Oral and Topical Therapy in Adult‐Onset Acne With Hyperpigmentation Concerns

A 28‐year‐old Indian female with no significant medical history presented with a 1‐month history of recurrent facial acne (CASS grade 3), affecting the forehead, bilateral cheeks, nose, and chin. She had been using over‐the‐counter cosmeceuticals prior to seeking dermatological consultation. Examination revealed multiple hyperpigmented follicular papules on the forehead, cheeks and chin, and closed comedones on the nose with postinflammatory hyperpigmentation across the face. There was also pre‐existing scaliness in the perioral and perinatal region. The patient reported low self‐esteem due to acne and acne‐induced hyperpigmentation. The patient was initiated on oral doxycycline 100 mg once daily alongside topical trifarotene, applied every other night for 1 week with a plan to increase to nightly application if tolerated. Trifarotene was selected due to extensive comedonal involvement, particularly on the nose requiring a faster clinical response, and the presence of post‐inflammatory hyperpigmentation, for which trifarotene has demonstrated efficacy. To enhance tolerability, trifarotene was applied after a layer of moisturizer, initially targeting acne‐affected areas before gradually extending to hyperpigmented regions.

**FIGURE 3:**
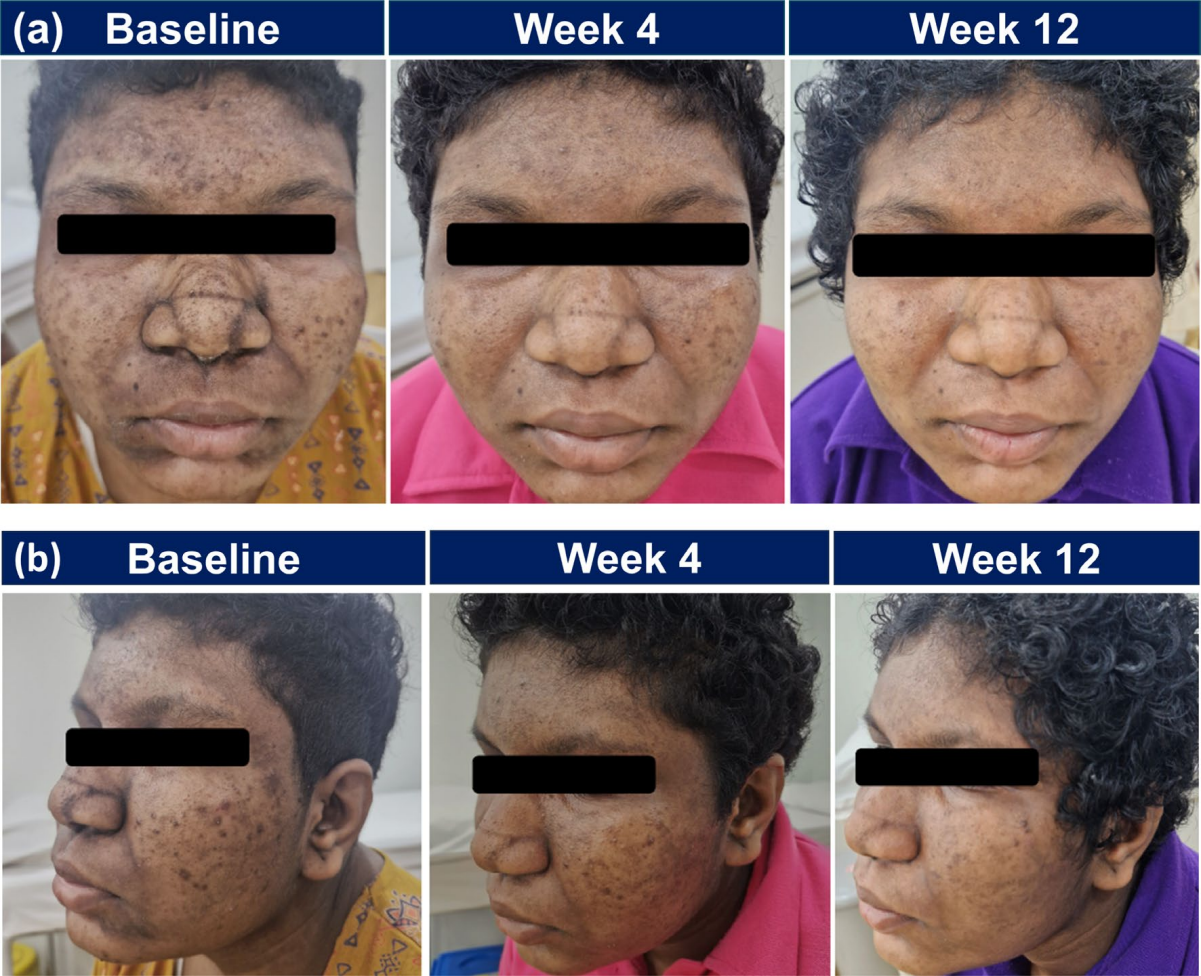
Clinical response in a patient with acne vulgaris and acne‐induced hyperpigmentation treated with trifarotene on face (a, b) for Case 3.

By week 4, visible improvement in acne lesions and pigmentation was noted, although trifarotene‐induced dryness required alternate‐night application to be continued. Further improvement was observed at week 12, with lighter skin tone and reduced hyperpigmentation (Figure 3). Gradual resolution of dryness and scaliness occurred with continued use of trifarotene and moisturizer. Oral doxycycline was discontinued, and the patient was continued on topical trifarotene monotherapy at the last follow‐up for maintenance.

This case illustrates how trifarotene can be used to manage active acne with acne‐induced hyperpigmentation, addressing both inflammatory lesions and post‐inflammatory hyperpigmentation. Its complementary effect when combined with oral antibiotics may contribute to improved treatment outcomes.

## 4 Discussion

The present advisory statements provide a structured framework for integrating trifarotene into the current acne management, addressing both facial and truncal involvement. Overall, the evidence and consensus reported herein reinforce trifarotene's notable benefits: early reduction in lesion counts, improvement in atrophic scarring and hyperpigmentation, and favorable tolerability [11, 12, 16, 18]. These findings are particularly relevant to the Malaysian clinical setting, where current guidelines provide limited coverage of trifarotene due to its unavailability in the market at the time of guideline development.

Multiple clinical trials, including the PERFECT series [16], DUAL [17], and Blume‐Peytavi et al. [18], highlighted trifarotene's efficacy across mild‐to‐moderate facial acne, with expansions into moderate‐to‐severe cases when used in combination with systemic agents. By selectively targeting RARγ receptors, trifarotene exerts comedolytic and anti‐inflammatory actions [10, 13], producing notable improvements observed as early as 2–4 weeks of therapy [16]. Evidence of rapid depigmenting effects and scar reduction aligns with panelists' observations in real‐world settings, where patients often prioritize both lesion clearance and acne‐induced sequelae [12]. The efficacy of trifarotene in improving acne‐induced hyperpigmentation is observed in clinical Case 3 (Figure 3).

Although first‐ and third‐generation retinoids demonstrate efficacy in the treatment of mild‐to‐moderate acne, the unique trunk‐focused data on trifarotene help bridge a gap in the current guidelines, which predominantly focus on facial lesions. Its low systemic absorption profile provides an added advantage for large‐area application, potentially minimizing the risk of systemic side effects [10]. Accordingly, the panel concurred that trifarotene represents a valuable option both as an initial therapy and for long‐term maintenance, particularly for truncal acne—an often underrecognized, yet clinically significant burden for many patients.

### 4.1 Safety and Tolerability

Consistent with other topical retinoids, trifarotene's most common adverse events include localized erythema, dryness, and irritation, peaking within the early weeks of therapy [18]. Strategies such as incremental application schedules (e.g., alternate‐day or brief‐contact therapy) and the use of gentle cleansers, non‐comedogenic moisturizers, and photoprotection have effectively minimized early dropouts due to skin irritation [12, 18, 43].

Panelists endorsed several supportive measures to mitigate irritation and improve adherence, including incremental application schedules and spot initiation approaches, where treatment is first applied to a small area to assess tolerability before gradually extending to broader field application. The cleanse‐treat‐moisturize‐photoprotect (CTMP) routine [32] outlined in Table 1, was also recommended to minimize irritation.

### 4.2 Special Populations and Combination Therapy

Clinical trials included a wide age range, including adolescent participants [11, 12, 16, 18], supporting trifarotene's efficacy in younger patients. Given that darker skin types are more susceptible to acne‐induced hyperpigmentation [12], the observed reductions in hyperpigmentation and scarring in clinical studies involving patients with darker skin [11, 12], suggest that trifarotene's benefits extend to this population. However, subgroup‐specific analyses and large‐scale real‐world data remain limited, highlighting the need for further research to better characterize treatment outcomes across diverse skin types. Data for use during pregnancy and lactation remain limited, prompting an advisory to discontinue trifarotene therapy if pregnancy is confirmed [19].

For severe or widespread acne, combination therapy with oral antibiotics (e.g., doxycycline) may offer complementary benefits, with early clinical experiences suggest synergistic effect on lesion resolution while maintaining a manageable tolerability profile [17]. This is further demonstrated in clinical Case 3 (Figure 3), where notable improvement was observed following combination therapy with doxycycline, which was subsequently discontinued after Week 12 while maintaining clinical benefit with trifarotene monotherapy.

A notable area of interest is the co‐administration of trifarotene with BPO. Laboratory data indicates that co‐application of tretinoin and BPO can lead to oxidative degradation of tretinoin [20]. While no specific stability studies are available for trifarotene when co‐administered with BPO, existing data suggest that it may be susceptible to similar degradation [44]. The advisory panel recommended applying BPO and retinoids at different times of the day (e.g., BPO in the morning, retinoids at night), a practice that could be extended to trifarotene to preserve stability and optimize efficacy.

### 4.3 Management of Acne Sequelae

Sequelae formed from primary active acne lesions, including atrophic acne scars and acne‐induced hyperpigmentation, are common. A meta‐analysis of 37 studies reported a 47% pooled prevalence of atrophic acne scarring in 24 649 acne patients, with increased risks in males, individuals with positive family history, and those with moderate‐to‐severe acne [45]. Acne‐induced hyperpigmentation most commonly occurs in individuals with darker skin tones, with a reported prevalence ranging from 45.5% to 87.2% [46, 47]. Studies suggest that inflammation plays a key role in the development of acne sequelae and early use of topical retinoids can inhibit inflammatory pathways, thereby reducing the onset of these acne‐induced sequelae [38, 48]. Trifarotene demonstrates potential in managing acne sequelae, including acne‐induced hyperpigmentation and acne‐induced scarring, with its use primarily supported as part of a comprehensive acne management approach rather than as a standalone therapy for sequelae [11, 12].

Adjunctive use of trifarotene with other modalities, such as lasers, chemical peels, and fillers, has been suggested to enhance scar management outcomes [25, 27, 28, 29]. Although prior studies highlight the benefits of combination approaches, robust evidence specific to trifarotene in this setting remains limited. Future larger‐scale studies are warranted to validate these combination regimens and optimize application protocols.

### 4.4 Patient Education and Long‐Term Outcomes

Adherence remains a key determinant of success in topical acne therapy [6]. Educational measures—ranging from multilingual materials to targeted counseling on skincare routines—consistently improve patient‐reported outcomes and reduce early dropouts [6, 39]. The emphasis on gradual onboarding, including incremental application, layering with moisturizers following an “open sandwich” regimen (applying moisturizer either before or after trifarotene), and close follow‐up plays a crucial role in enhancing adherence. Setting realistic expectations of 8–12 weeks for noticeable improvement can promote patient engagement and sustained use—key factors in managing chronic, relapsing conditions like acne.

### 4.5 Limitations and Future Directions

Although the advisory statements are grounded in robust evidence and expert consensus, there remain important evidence gaps. Limited head‐to‐head trials have compared trifarotene with other topical retinoids (e.g., adapalene, tretinoin, tazarotene), particularly in truncal acne and combination regimens. Uncertainties remain regarding trifarotene's role in acne‐induced erythema, stable acne scars, and its synergy with BPO or energy‐based devices. Real‐world studies encompassing diverse patient populations—including those with comorbidities, prior retinoid intolerance, or postpartum status—are needed to improve the external validity of current recommendations. Future research should further explore patient preferences and adherence for various vehicle formulations and the potential role of digital tools (e.g., teledermatology platforms) in optimizing adherence and outcomes.

## 5 Conclusion

These consensus‐based advisory statements highlight trifarotene's emerging role in acne management, offering an additional therapeutic option to address persistent unmet needs, including acne‐induced scarring and hyperpigmentation. By providing practical recommendations on patient selection, application techniques, side‐effect mitigation, and combination strategies, this paper supports the integration of trifarotene into routine clinical practice across dermatology, primary care, and aesthetic settings. As real‐world evidence accumulates and comparative research expands, particularly in diverse patient populations, greater clarity on trifarotene's potential in acne management is anticipated. These insights will contribute to a better understanding of the role of trifarotene in acne management.

## Author Contributions

A.M.A., K.K., and W.T.P. conceptualized the study. A.M.A. drafted the first draft of the manuscript. A.M.A., P.W.B.C., B.T.Y.T., E.W.Y.Y., C.C.C., S.H.F., K.N.H., Z.K., J.J.T., W.C.T., K.K., and W.T.P. contributed to the acquisition of data, reviewed and provided critical feedback on the manuscript, and participated in the revision and finalization of the submitted version.

## Funding

This work was supported by Galderma.

## Ethics Statement

The authors confirm that the ethical policies of the journal, as noted on the journal's author guidelines page, have been adhered to. No ethical approval was required.

## Consent

Written informed consent was obtained from the patients for publication of their clinical information and images. The patients were informed that their identities would remain confidential and that all efforts would be made to preserve their anonymity.

## Conflicts of Interest

Azura Mohd Affandi has received honoraria for serving as speaker and/or advisor for AbbVie, Beiersdorf, Boehringer Ingelheim, DKSH, Galderma, Glenmark, GSK, Hyphens, Johnson & Johnson, Loreal, Menarini, Novartis, Pfizer, Sanofi, Livemed, Taisho, and ZP Therapeutics. Peter Wee Beng Ch'ng has received honoraria for serving as trainer, speaker and/or advisor for AbbVie, Beiersdorf, Boehringer Ingelheim, DKSH, Galderma, Glenmark, GSK, Hyphens, Johnson & Johnson, Loreal, Menarini, Novartis, Pfizer, Sanofi, Taisho, ZP Therapeutics, Sunpharma, Merz, Lutronic, Neoasia, and Vanguard. Benji Tze Yuen Teoh has received honoraria for serving as speaker and/or advisor for AbbVie, Beiersdorf, Boehringer Ingelheim, DKSH, Galderma, GSK, Johnson & Johnson, Loreal, Menarini, Novartis, Pfizer, Sanofi, Taisho, and ZP Therapeutics. Evelyn Wen Yee Yap serves as a consultant for AnaptysBio and has received research funding as a principal investigator from AbbVie, AnaptysBio, Boehringer Ingelheim, Novartis, and La Roche‐Posay. Chin Chwen Ch'ng has received honoraria for serving as speaker and/or advisor for AbbVie, Beiersdorf, DKSH, Galderma, GSK, Johnson & Johnson, Loreal, Menarini, Novartis, Pfizer, Sanofi, Hyphens, and ZP Therapeutics. Seow Hoong Foo has received honoraria for serving as speaker and/or advisor for Galderma, Loreal, Menarini, and Taisho. Kang Nien How has received honoraria for serving as speaker and/or advisor for Beiersdorf, Elogio Asia, Galderma, Loreal, Menarini, Neoasia, Novartis, Pfizer, Sanofi, and Vangard Aesthetic. Zhenli Kwan has received honoraria for serving as trainer, speaker and/or advisor for AbbVie, Beiersdorf, Boehringer Ingelheim, DKSH, Galderma, Hyphens, Loreal, Menarini, Novartis, Pfizer, Sanofi, Taisho, ZP Therapeutics, and Sunpharma. Jyh Jong Tang has received honoraria for serving as speaker and/or advisor for AbbVie, Boehringer Ingelheim, Galderma, Glenmark, Hyphens, Johnson & Johnson, Novartis, Pfizer, Taisho, and ZP Therapeutics. Wooi Chiang Tan has received honoraria for serving as speaker and/or advisor for AbbVie, Beiersdorf, Boehringer Ingelheim, DKSH, Galderma, GSK, Johnson & Johnson, Loreal, Menarini, Novartis, Pfizer, Sanofi, Livemed, Taisho, and ZP Therapeutics. All advisory panel members received honoraria from Galderma. The funding source had no role in the analysis of the study and interpretation of the results. Khen Kon and Wei Thian Poh are employees of Galderma.

## Supporting information


**Appendix S1:** jocd70625‐sup‐0001‐supinfo.docx.

## Data Availability

The data that supports the findings of this study are available in the Supporting Information of this article.
