# Supplementary material for: Bridging Gaps in Malaysian Acne Vulgaris Guidelines: Advisory Statements on Trifarotene for Facial and Truncal Acne
Source: J Cosmet Dermatol. 2025 Dec 28;25(1):e70625. doi: 10.1111/jocd.70625 (PMC12745670; doi:10.1111/jocd.70625)
Supplement: Supplementary file 1 — Appendix S1: jocd70625‐sup‐0001‐supinfo.docx. [file JOCD-25-e70625-s001.docx]

**Appendix**

**Table of Contents**

[Table A1. Pre-specified Framework 2](#_Toc195110321)

[Figure A1. PRISMA flow diagram 3](#_Toc195110322)

[Table A2. Summary of Main Clinical Trials for Trifarotene 4](#_Toc195110323)

[Table A3. Summary of Evidence Extracted from Eligible Studies for Advisory Statement Development 7](#_Toc195110324)

[Table A4. Pre-Advisory Meeting Survey, Agreement Rating and Feedback / Comment on Clinical Advisory Statement 22](#_Toc195110325)

[References 25](#_Toc195110326)

## Table A1. Pre-specified Framework

| **No.** | **Key Clinical Advisory Statement** | **Detailed information for Clinical Advisory Statement** |
| --- | --- | --- |
| **1** | **Trifarotene's Role in Acne Treatment** | Define trifarotene's position within current acne treatment guidelines |
|  |  | Highlight the advantages of trifarotene over other topical retinoids |
| **2** | **Patient Selection Criteria** | Identify ideal candidates for trifarotene therapy |
|  |  | Emphasise effectiveness for both facial and truncal acne |
| **3** | **Dosage and Administration** | Provide recommended dosing regimens and application techniques |
|  |  | Offer guidance on treatment duration and patient instructions |
| **4** | **Combination Therapy** | Recommend safe and effective combinations with other acne treatments |
|  |  | Outline benefits and protocols for combination therapy |
| **5** | **Management of Side Effects** | Address common adverse effects and strategies for their management |
|  |  | Suggest monitoring protocols to ensure patient safety |
| **6** | **Use in Special Populations** | Guidance on use in adolescents, pregnant women, and patients with comorbidities |
|  |  | Highlight any precautions or contraindications |
| **7** | **Patient Education and Adherence** | Strategies to improve adherence and set realistic expectations |
|  |  | Importance of patient counseling and support materials |
| **8** | **Addressing Unmet Needs** | Trifarotene's effectiveness in treating truncal acne and challenging cases |
|  |  | How trifarotene fills gaps in current acne management practices |

## Figure A1. PRISMA flow diagram

**Identification of Eligible Studies**

Studies identified through other sources

Google scholar (n = 100)

Studies identified from databases

PubMed (n = 67)

Cochrane library (n=33)

**Identification**

Duplicates removed (n = 72)

Reports assessed for eligibility.

(n = 24)

Additional publication added from expert recommendation
(n = 2)

Studies sought for retrieval.

(n = 47)

Studies screened.

(n = 128)

Studies included in review.

(n = 26)

Removed after examining title and abstract (n = 81)

- Do not contain management of acne vulgaris (n = 47)

- Clinical trial registration (n = 15)

- Full publication not found (n = 17)

- Publication not in English (n = 2)

**Screening**

Removed after examining the content (n = 23)

- Do not contain management of acne vulgaris (n = 8)

- Insignificant articles reviews (n = 15)

**Included**

## Table A2. Summary of Main Clinical Trials for Trifarotene

| **Trial Name** | **Phase** | **Objective** | **Design** | **Primary Endpoints** | **Key Results** | **Safety Profile** |
| --- | --- | --- | --- | --- | --- | --- |
| PERFECT 1  (2019) ^1^ | 3 | Evaluate the safety and efficacy of trifarotene for moderate facial and truncal acne | 12-week, randomised, double-blind, vehicle-controlled  Age: 9 years and older (inclusion criteria for trunk acne optional for 9-11 years) | Primary: Facial acne IGA success, absolute change in IL and NIL count at Week 12  Secondary: Truncal PGA success, absolute change in IL and NIL count at Week 12 | **Primary:**  Facial acne IGA success:  29.4% in trifarotene group vs. 19.5% in vehicle group (*P*<0.001); rate of success significant starting at week 4.  Absolute change in facial lesion count at Week 12: -19.0% trifarotene vs. -15.4% vehicle (*P*<0.001) for IL; -25.0% trifarotene vs. -17.9% vehicle (*P*<0.001) for NIL; reduction in facial lesion count as early as week 2.  **Secondary:**  PGA success: 35.7% trifarotene vs. 25.0% vehicle (P<0.001) rate of success significant starting at week 8.  Absolute change in truncal lesion count at Week 12: -21.4% trifarotene vs. -18.8% vehicle (*P*<0.001) for IL; -21.9% trifarotene vs. -17.8% vehicle (*P*<0.001) for NIL; reduction in truncal lesion count as early as week 4. | Mostly mild-to-moderate irritation  Maximum severity at week 1 for face, weeks 2-4 for trunk |
| PERFECT 2  (2019) ^1^ | 3 | Evaluate the safety and efficacy of trifarotene for moderate facial and truncal acne | 12-week, randomised, double-blind, vehicle-controlled  Age: 9 years and older (inclusion criteria for trunk acne optional for 9-11 years) | Primary: Facial acne IGA success, absolute change in IL and NIL count at Week 12  Secondary: Truncal PGA success, absolute change in IL and NIL count at Week 12 | **Primary:**  IGA success: 42.3% trifarotene vs. 25.7% vehicle (*P*<0.001); [rate of success significant starting at week 8.  Absolute change in facial lesion count at Week 12: -24.2% trifarotene vs. -18.7% vehicle (*P*<0.001) for IL; -30.1% trifarotene vs. -21.6% vehicle (*P*<0.001) for NIL; reduction in facial lesion count as early as week 1.  **Secondary:**  PGA success: 42.6% trifarotene vs. 29.9% vehicle (P<0.001); rate of success significant starting at week 8.  Absolute change in truncal lesion count at Week 12: -25.5% trifarotene vs. 19.8% vehicle (*P*<0.001) for IL; -25.9% trifarotene vs. -20.8% vehicle (*P*<0.001) for NIL; reduction in truncal lesion count as early as week 2. | Mostly mild-to-moderate irritation  Maximum severity at week 1 for face, weeks 2-4 for trunk |
| Blume-Peytavi et al. (2020) ^2^ | 4 | Evaluate long-term safety and efficacy of trifarotene in moderate facial and truncal acne (52 weeks) | 52-week, multicentre, open-label study  Age: 9 years and older (inclusion criteria for trunk acne optional for 9-11 years) | Primary: Local tolerability signs and symptoms, and TEAEs at Week 12, 20, 26, 38, 52  Secondary: IGA and PGA success rates at Week 12, 20, 26, 38, 52, quality of life improvements at Week 12, 26, 52 | **Efficacy:**  IGA success rate: 26.6% at Week 12 to 65.1% at Week 52.  PGA success rate: 38.6% at Week 12 to 66.9% at Week 52.  Improved quality of life. | Mild-to-moderate cutaneous irritation (early), TEAEs in 12.6%, no serious AEs |
| DUAL  (2022) ^3^ | 4 | Evaluate the efficacy and safety of trifarotene with doxycycline for severe facial acne | 12-week, double-blind, placebo-controlled  Age: 12 years and older | Primary: absolute change in total lesion count at Week 12  Secondary: changes in IL and NIL counts, IGA success | **Primary:**  Absolute change in total lesion counts at Week 12: -69.1 T+D vs. -48.1 V+P (*P*<0.0001).  **Secondary:**  Change in lesion count at Week 12: -29.4 T+D vs. -19.5 V+P for IL, and -39.5 T+D vs. -28.2 for NIL (*P*<0.0001).  IGA success: 31.7% T+D vs. 15.8% V+P (*P*=0.01). | Mild, transient irritation; TEAEs in 13.5% in the T+D group and 15.9% in the V+P group. |
| START  (2023) ^4^ | 4 | Assess trifarotene's effect on atrophic acne scars | 24-week, split-face, vehicle-controlled, double blind  Age: 17 to 34 years | Primary: absolute change in total atrophic acne scar count at Week 24  Secondary: Changes in 2-4mm & >4mm and total atrophic scar count from baseline to week 20, SGA & SGA success, percent change in AV lesion, IGA and IGA success | **Primary**:  Absolute change in total atrophic acne scar count at Week 24: -5.9 in trifarotene-treated area vs. -2.7 in vehicle-treated area at Week 24 (*P*<0.0001); difference noted as early as week 2.  **Secondary:**  SGA success: 14.9% trifarotene vs. 5.0% vehicle *(P*<0.05) at Week 12 and improved through Week 24 (31.3% vs 8.1%, *P*<0.001).  IGA success: 63.6% trifarotene vs. 31.3% vehicle, (*P*<0.0001). | Mild-to-moderate side effects; TEAEs in 5.8% (trifarotene) |
| LEAP  (2024) ^5^ | 4 | Assess the efficacy and safety of trifarotene for moderate AV and AIH on the face | 24-week, parallel-group, vehicle-controlled  Age: 13 to 35 years | Primary: absolute change in ODS score at week 24  Secondary: percent change in ODS at week 24, absolute/percent change in ODS at weeks 12, 16, 20, average AIH lesion size, PAHPI score, AV lesion count, IGA success | **Primary:**  Absolute change in ODS score: -34.4% in trifarotene group vs -23.6% in vehicle group from baseline at Week 12 (*P*=0.03);NS at week 16 and 24.  **Secondary:**  Change in PAHPI score -18.9% in trifarotene group vs -11.3 in vehicle group at Week 24 from baseline (*P*<0.01); NS at Week 12 and 16.  Change in AV lesion count: -72.0% in the trifarotene group vs -62.8% in the vehicle group (*P* < 0.05) at Week 24.  IGA success: 61.1% in trifarotene group vs 39.4% in vehicle group (*P*<0.05) at Week 24.  Note: Marked improvement in focal facial erythema noted in light-skinned individuals (although study not designed to assess erythema). | Mild, low incidence of TEAEs (16.7% in trifarotene group) |

Abbreviations: AIH: Acne-Induced Hyperpigmentation; AV: Acne Vulgaris; IGA: Investigator’s Global Assessment; IL: Inflammatory Lesion; NIL: Non-Inflammatory Lesion; ODS: Overall Disease Severity; PAHPI: Post-Acne Hyperpigmentation Index; PGA: Physician’s Global Assessment; SGA: Subject’s Global Assessment; TEAEs: Treatment-Emergent Adverse Events; T+D: Trifarotene + Doxycycline; V+P: Vehicle + Placebo.

Note: A *P*-value less than 0.05 is considered statistically significant.

## Table A3. Summary of Evidence Extracted from Eligible Studies for Advisory Statement Development

| **Statement** | **Author, year** | **Evidence extracted from cited articles*** |
| --- | --- | --- |
| **Statement 1. Integrate Trifarotene into Existing Acne Management Algorithms** | | |
| Novel Mechanism | Aubert et al. (2018) ^6^ | We described the pharmacology of trifarotene, **the first potent RARc-selective agonist entering clinical trials.** The molecule was designed to present good metabolic stability in cultured keratinocytes, while being rapidly metabolised in human liver microsomes.  Trifarotene regulated the expression of genes involved in known retinoid-induced pathways, including retinoid metabolism (CYP26A1, STRA6, DHRS9, CRABP2), epidermal differentiation (KRT4, ELF3, PPARD, ID1), proliferation (FOSL1, P2RY2, RIT1, CCNG2, ZBTB20, FGFR2, BTC) and response to stress (S100A9, GPX2, IL1RN, CXCR2, TYMS, F3). Interestingly, **three retinoid-modulated pathways were identified that have not yet been described:** **proteolysis** (PRSS27, KLK6, KLK8, KLK10, CTSD, SERPINA12, MME) (Fig. 4b), **transport/skin hydration** (PADI1, AQP3, RHCG, ATP11B) (Fig. 4c) **and cell adhesion** (PRRG4, EFHD2, CHL1, SPON1, DST) (Fig. 4d). For the proteolysis pathway, the induction of KLK6, KLK8 and KLK10 expression was confirmed by immunohistochemistry (Fig. 4e).” |
|  | Thoreau et al. (2018) ^7^ | In conclusion, **determination of the structure of known RARγ-selective agonist CD437 revealed an unprecedented and isotype-specific pocket in the RARγ ligand binding domain.** Optimisation of a series of a novel triaryl compounds directed at this pocket, led to the identification of 15b (CD5789/Trifarotene), which combines potency, selectivity and high metabolic instability and which is currently undergoing clinical trials in the topical treatment of acne.” |
|  | Dreno et al. (2021) ^8^ | A total of 67 genes were uniquely affected by trifarotene–these genes did not appear in the spontaneously resolving acne lesion signature (Figure 2A, Supplementary Table 3). A majority of these genes were upregulated in acne lesions (as compared to non-involved skin) and were concomitantly downregulated by trifarotene treatment only (Figure 2B). **The most significantly down-regulated genes included the chemokines CXCL13** (Fold-Change FC = −23.5, FDR = 0.0032) and XCL1 (FC = −2.02, FDR = 0.013), the phosphoglycoprotein osteopontin (SPP1, FC = −28.2, FDR = 0.0022) and the matrix metalloproteinases MMP12 and MMP13 (FC = −11.13, FDR = 0.061; FC = −4.3, FDR = 0.029, respectively). **These genes influence inflammatory cell infiltration (including CXCL13, XCL1, and SPP1/osteopontin) and extracellular matrix reorganisation (MMP12 and MMP13)** (Supplementary Table 3). IPA analysis of the 67 genes (Figure 2C) revealed that an upregulation of pathways associated with inflammatory response, leukocyte migration, neutrophil movement, and T cell migration seen in the papule was reversed following trifarotene treatment (Figure 2C, lower panel, Supplementary Table 4 for statistical results and list of molecules involved in each pathway). |
| Pharmacokinetics | Aubert et al. (2018) ^6^ | Trifarotene was **stable in human keratinocytes for more than 24 hours** and very **rapidly metabolised in human liver microsomes with a half-life (t1/2) of 5 minutes.** |
| Clinical Trials | Tan et al. (2019) ^1^ | For the 1214 patients treated with trifarotene and 1206 treated with vehicle, the **week 12 facial success rates according to the IGA were 29.4% in PERFECT 1 and 42.3% in PERFECT 2** (vs 19.5% and 25.7% for vehicle [P <.001]); trifarotene had **statistically significant superior success rates** at week 4 (PERFECT 1) and week 8 (PERFECT 2). Trifarotene treatment achieved **significantly superior reductions in facial lesion counts** as well, with statistical differences **apparent as early as weeks 2** and 1: with trifarotene treatment, **the mean absolute inflammatory lesion counts were reduced by 19.0 and 24.2** (vs by 15.4 and 18.7 with vehicle [P < .001]) and **the mean absolute noninflammatory lesion counts were reduced by 25.0 and 30.1** (vs by 17.9 and 21.6 with vehicle [P < .001]). |
|  | Del Rosso et al. (2022) ^3^ | There was a greater mean absolute change in total lesion counts from baseline to Week 12 (T+D -69.1 vs. -48.1 V+P), with a significant treatment difference (LSMean -21.0, P <0.0001) in favour of T+D. By Week 12, **the percent change in total lesion count was -67.0 percent in the T+D group compared to -45.5 percent in the V+P group**. As shown in Figure 1, reductions in individual lesion types were also superior in the T+D group compared to V+P, with a between treatment LSMean difference of -10.0, P<0.0001 for inflammatory lesions and LSMean difference of -11.3, p<0.0001 for non-inflammatory lesions.  The proportion of subjects achieving IGA success at Week 12 was 31.7 percent in the T+D group vs 15.8 percent in the V+P group, difference 15.9 percent (P=<0.05). |
|  | Schleicher et al. (2023) ^4^ | A **significantly superior reduction in atrophic scar count occurred on trifarotene-treated sides** **of the face** compared to vehicle-treated sides. At week 24, the mean absolute change from baseline was − 6.2 ± 5.6 on the trifarotene-treated area compared to − 2.8 ± 3.9 on the vehicle area (Δ − 3.2, P < 0.0001). This was primarily due to a greater amount of reduction in 2–4 mm atrophic acne scars. As shown, **there was a difference between treated sides as early as week 2** (P = 0.001), which continued at week 4 (P = 0.007) and weeks 8 through 20 (P < 0.0001). The total scar counts were similar between groups at baseline (Table 1), but **by week 24, the total scar count was 5.4 ± 5.6 for trifarotene-treated areas and 9.1 ± 8.6 for vehicle-treated areas (percent reductions of 55.2% and 29.9%, respectively)**.  The SGA success rate was higher in the trifarotene side at week 12 (14.9% vs 5.0%, P <0.05) and improved through week 24 (31.3% vs 8.1%, P <0.001). Similarly, at week 24, the IGA success rate was higher with trifarotene (63.6% vs 31.3%, P <0.0001) along with reductions in total (70% vs 45%) and inflammatory (76% vs 48%) lesion counts. |
|  | Alexis et al. (2024) ^5^ | Trifarotene 50 μg/g cream **improved significantly from baseline in ODS score** versus vehicle (−1.6 vs. −1.1, P = 0.03) **at Week 12, but scores were comparable between groups at Week 24** (primary endpoint).  Trifarotene had **a better reduction in PAHPI score at Week 24** (−18.9% vs. −11.3% vehicle, P < 0.01). **Lesion count reductions were higher with trifarotene at Week 12 versus vehicle** (P < 0.001) and at **Week 24** (P < 0.05), as **were IGA success rates versus vehicle at Weeks 12** (P < 0.05) **and 24** (P < 0.05). |
|  | Blume-Pytavi et al. (2020) ^2^ | At Week 12, IGA and PGA success rates were 26.6% and 38.6%, respectively. **Success rates increased to 65.1% and 66.9%, respectively at Week 52.** Overall success (both IGA and PGA success in the same patient) was 57.9% at Week 52. |
| Comparison gap | Shergil et al. (2024) ^9^ | The objective of this systematic literature review and meta-analysis of outcomes from phase 3 clinical trials was to compare the efficacy between clascoterone, trifarotene, and tazarotene for the treatment of acne vulgaris to guide the clinical management of acne vulgaris treatment. The analyses showed robust differences favouring interventions for ILC (MD: − 11.5; 95% CI: − 14.4, − 8.6; Fig. 2a) and NILC (MD:−12.3; 95% CI:−15.2,−9.3; Fig. 2b) at week 12. However, tests for subgroup differences did not identify significant differences between clascoterone, trifarotene, and tazarotene for ILC (MD:−12.8,−11.2, and−10.1, respectively; P = 0.82; Fig. 2a) or NILC (MD: − 11.6, − 13.9, and−12.8, respectively; P=0.81; Fig. 2b).  The OR for the rate of treatment success similarly indicated favourable treatment efficacy for the interventions at week 12 (OR: 2.1; 95% CI: 1.8, 2.5). However, no significant differences were observed between clascoterone, trifarotene, and tazarotene (OR: 2.9, 1.9, and 2.1, respectively; P=0.16; Fig. 3). |
|  | Aubert et al. (2018) ^6^ | Importantly, topically applied trifarotene was active in ex vivo cultured human skin, with a stratum corneum barrier close to human skin in vivo. Interestingly, **in this assay trifarotene was threefold more potent than tazarotene, which is in agreement with the demonstrated overall higher in vitro potency of trifarotene compared with tazarotenic acid**, the active metabolite of the prodrug tazarotene. |
| Algorithm Positioning | Tan et al. (2019) ^1^ | The eligibility criteria were patient age 9 years and older, **moderate facial acne** (defined as an Investigator’s Global Assessment [IGA] score of 3 on the face [≥20 inflammatory lesions and ≥25 noninflammatory lesions]), and **moderate truncal acne** (defined as a Physician’s Global Assessment [PGA] score of 3 at screening and baseline [≥20 inflammatory lesions and 20 to <100 noninflammatory lesions on the areas of the trunk reachable for self-application]).  Treatment: Trifarotene 50mug/g cream for facial and truncal acne Comparison: Vehicle cream for facial and truncal acne |
|  | Del Rosso et al. (2022) ^3^ | Eligible patients were aged 12 years or older with **severe facial acne** defined as an IGA score of 4 on the face (≥20 in­flammatory lesions, 30 to 120 non-in­flammatory lesions, and ≤4 nodules).  Treatment: trifarotene cream 50mug/g + doxycycline 120mg  Comparison: vehicle cream + placebo |
| Long-Term Potential | Schleicher et al. (2023) ^4^ | Refer to Statement 1: Clinical Trials |
|  | Alexis et al. (2024) ^5^ | Refer to Statement 1: Clinical Trials |
|  | Tan et al. (2019) ^1^ | Refer to Statement 1: Clinical Trials |
|  | Blume-Pytavi et al. (2020) ^2^ | Refer to Statement 1: Clinical Trials |
|  | Del Rosso et al. (2022) ^3^ | Refer to Statement 1: Clinical Trials |
| Drug Interaction | Wagner et al. (2020) ^10^ | QTC investigations did not show any risk of cardiovascular health issues; **trifarotene did not reduce the systemic exposure to oral contraceptives such as** levonorgestrel/ethinyl estradiol. |
|  | Trifarotene – Malaysia Product Information. (2022) ^11^ | A clinical drug-drug interaction study has shown **that topical application of trifarotene did not affect the circulating concentrations of hormonal contraceptives** (ethinylestradiol and levonorgestrel) administered by oral route. |
| Guideline Gap | Malaysia MoH. (2022) ^12^ | Section 5.2.5 Topical trifarotene (Page 17) |
| **Statement 2. Patient Selection Criteria** | | |
| Efficacy Across Acne Severity | Tan et al. (2019) ^1^ | Refer to Statement 1: Algorithm Positioning |
|  | Del Rosso et al. (2022) ^3^ | Refer to statement 1: Algorithm Positioning  **Trifarotene + Doxycycline was demonstrated to be safe and efficacious** as a treatment option for patients with severe acne. |
| Hyperpigmentation / Scarring | Schleicher et al. (2023) ^4^ | Refer to Statement 1: Clinical Trials  A **significantly superior reduction in atrophic scar count occurred on trifarotene-treated sides** **of the face** compared to vehicle-treated sides. At week 24, the mean absolute change from baseline was − 6.2 ± 5.6 on the trifarotene-treated area compared to − 2.8 ± 3.9 on the vehicle area (Δ − 3.2, P < 0.0001). This was primarily due to a greater amount of reduction in 2–4 mm atrophic acne scars. As shown, **there was a difference between treated sides as early as week 2** (P = 0.001), which continued at week 4 (P = 0.007) and weeks 8 through 20 (P < 0.0001). The total scar counts were similar between groups at baseline (Table 1), but **by week 24, the total scar count was 5.4 ± 5.6 for trifarotene-treated areas and 9.1 ± 8.6 for vehicle-treated areas (percent reductions of 55.2% and 29.9%, respectively)**. |
|  | Alexis et al. (2024) ^5^ | Refer to Statement 1: Clinical Trials  Trifarotene had **a better reduction in PAHPI score at Week 24** (−18.9% vs. −11.3% vehicle, P < 0.01). **Lesion count reductions were higher with trifarotene at Week 12 versus vehicle** (P < 0.001) and at **Week 24** (P < 0.05), as **were IGA success rates versus vehicle at Weeks 12** (P < 0.05) **and 24** (P < 0.05). |
|  | Aubert et al. (2018) ^6^ | Unlike other retinoids, and potentially due to a weaker penetration in hyperkeratotic tail skin, adapalene 0.1% showed no significant depigmenting activity after 6 weeks of topical application on the SKH2 mouse tail (Fig. 3b). In contrast, **trifarotene and ATRA showed significant depigmenting activity at 001%** (11 and 1, respectively, on the pigmentation score at day 43). After UVR induction, in the same mouse model, the antipigmenting activity of trifarotene was again highly significant. |
| Exclusions | Reynolds et al. (2024) ^13^ | Oral isotretinoin, or 13-cis-retinoic acid, is the only FDA-approved treatment for severe recalcitrant nodular acne vulgaris since 1982. In a RCT of 33 patients with treatment-resistant cystic and conglobate acne**, mean number of cystic lesions decreased by 17% and 33% at 1 and 2 months of isotretinoin treatment, but increased by 33% and 58% at 1 and 2 months of placebo**. |
|  | Thiboutot et al. (2017) ^14^ | Consensus recommendation 3: **oral isotretinoin should be first-line therapy for very severe (cystic and conglobate) acne.** Isotretinoin is a highly efficacious acne treatment, proven to clear acne lesions, including nodules and cysts, and achieve a prolonged remission period. |
| Clinical Assessment | Malaysia MoH. (2022) ^12^ | The Dermatological Society of Singapore and the first edition of Malaysian MoH CPG on Management of Acne advocate the use of Comprehensive Acne Severity Scale (CASS) for evaluating acne severity. CASS [modification of an Investigator Global Assessment (IGA) of Acne Severity] is a validated tool. It correlates strongly with the Leeds technique for face (r=0.82), chest (r=0.85) and back (r=0.87). |
| **Statement 3. Dosage and Administration** | | |
| Consistency and Long-Term Use | Tan et al. (2019) ^1^ | Assessment of the efficacy and safety of CD5789 (trifarotene) 50μg/g cream applied once daily for 12 weeks in subjects with acne vulgaris. In the intervention arm, CD5789 (trifarotene) 50µg/g cream was applied once daily for 12 weeks. |
|  | Aubert et al. (2018) ^6^ | Refer to Statement 1: Novel Mechanism |
| Addressing Skin Barrier Needs | Tan et al. (2019) ^1^ | **Local tolerability**   - Local irritation related to trifarotene cream was transient and consistent with the known pattern of topical retinoid dermatitis (Fig 3); **tolerability was better on the trunk than on the face**. Local tolerability signs and symptoms related to trifarotene cream included **erythema, scaling, dryness, and stinging/burning**. These were mostly mild to moderate by investigator assessment, with few being severe. For facial acne, a worst postbaseline score of moderate local tolerability signs and symptoms compared with baseline was reported for up to 33.2% of patients (in PERFECT 1: erythema, 23.7%; scaling, 21.4%; dryness, 23.0%; and stinging/burning, 16.3%; in PERFECT 2: erythema, 33.2%; scaling, 32.9%; dryness, 36.4%; and stinging/burning, 24.9%) and severe for up to 10.0% of patients (in PERFECT 1: erythema, 2.5%; scaling, 2.9%; dryness, 2.5%; and stinging/burning, 4.2%; in PERFECT 2: erythema, 10.0%; scaling, 6.8%; dryness, 7.1%; and stinging/burning, 7.6%). On the trunk, the corresponding percentages of worst postbaseline local tolerability signs and symptoms were moderate for up to 23.2% of patients (in PERFECT 1: erythema, 14.6%; scaling, 10.8%; dryness, 11.3%; and stinging/burning, 9.0%; in PERFECT 2: erythema, 23.2%; scaling, 16.7%; dryness, 20.9%; and stinging/burning, 12.9%) and severe for up to 7.2% of patients (in PERFECT 1: erythema, 3.3%; scaling, 0.3%; dryness, 1.2%; and stinging/burning, 3.0%; in PERFECT 2: erythema, 7.2%; scaling, 3.0%; dryness, 2.5%; and stinging/burning, 5.7%). **The scores reached maximum severity at week 1 for the face and at weeks 2 to 4 on the trunk; after these time points, scores diminished**. |
|  | Blume-Pytavi et al. (2020) ^2^ | The most common cutaneous trifarotene-related TEAEs were pruritus [21 (4.6%) patients], irritation [19 (4.2%) patients] and sunburn [8 (1.8%) patients]; these were mainly observed on treated areas and were of **mild severity.** Severe trifarotene-related TEAEs occurred in 3 (0.7%) different patients: application site irritation, pruritus and erythema.  As observed with other retinoids, the majority **of these local adverse events occurred during the first weeks of treatment, subsiding thereafter; all were mild or moderate in intensity.**  A total of 16 (3.5%) patients discontinued the study due to TEAEs, among which 13 (2.9%) discontinued due to related TEAEs. The latter were considered AESIs – 10 events were skin irritation, and three events were worsening of acne. All occurred on the face and trunk during the first 3 months were related and non-serious. |
| Real-World Insights | Johnson et al. (2020) ^15^ | This case series illustrating the treatment of facial and truncal acne with trifarotene 50 lg/g cream, in the form of real-world data, describes high overall satisfaction and excellent tolerability to support the use of this new retinoid molecule in the treatment of acne vulgaris on both the face and trunk.  **All subjects indicated being overall satisfied with the trifarotene 50 lg/g topical treatment for both the face and trunk, satisfied with how easy it was to use and satisfied with the time the treatment took to work**. |
| **Statement 4. Combination Therapy (Other Topical Agents & Systemic Agents for Acne)** | | |
| Oral Antibiotics | Del Rosso et al. (2022) ^3^ | The study evaluated the efficacy and safety of trifarotene plus oral doxycycline in acne. The absolute change in lesion counts from baseline were: -69.1 T+D versus -48.1 V+P for total lesions, -29.4 T+D versus -19.5 V+P for in­flammatory lesions, and -39.5 T+D versus -28.2 for non-in­flammatory lesions (P<0.0001 for all). Success was achieved by 31.7 percent of subjects in the T+D group versus 15.8 percent in the V+P group (P=0.0107). Combination therapy with a topical retinoid and oral antibiotic is an efficacious therapy for severe acne, with a rapid onset of action and good safety and tolerability. |
| Benzoyl Peroxide (BPO) | Martin et al. (1998) ^16^ | With and without exposure to light, adapalene exhibits a remarkable stability whereas tretinoin is very sensitive to light and oxidation. **The combination of benzoyl peroxide and light results in more than 50% degradation of tretinoin in about 2 h and 95% in 24 h.** |
| Combined Approach | Grobel et al. (2018) ^17^ | Of note, **benzoyl peroxide can inactivate tretinoin and make it less stable and thus should not be used at the same time**. It is better to use benzoyl peroxide in the morning and tretinoin at night. |
| Use with Oral Isotretinoin | Miranti SM. (2024) ^18^ | A total of 12 patients completed 24.3 (6.7) weeks (mean [standard deviation]) of oral isotretinoin (cumulative dose: 184.6 [75.1] mg/kg) and 13.0 (6.7) months of post-isotretinoin tazarotene 0.045%. Photographs of improvements with oral isotretinoin and post-isotretinoin tazarotene 0.045% are shown in Figure 1. **No patients relapsed and all showed subjective visual improvements** in acne-related scarring with topical tazarotene maintenance treatment. |
|  | Vender R. (2012) ^19^ | Subjects successfully completed a treatment of acne vulgaris with oral isotretinoin (120–150 mg/kg/course) and were randomised to tretinoin 0.04% microsphere gel and vehicle gel. Overall, there was a trend to the efficacy of tretinoin 0.04% microsphere gel to the prevention of recurrent acne after isotretinoin use in male patients over 18 years old over a six-month period. **A 38.7% lower lesion count was observed in the tretinoin 0.04% microsphere gel group**. The discrepancy between the lesion count scores and the ISGA assessment at week 24 can be explained by the fact that the ISGA is an overall global assessment and is performed before the lesion counts as not to bias the evaluator. |
|  | Trunchelo et al. (2015) ^20^ | This is a prospective, randomised, double-blind and vehicle-controlled study of 30 patients with acne previously treated with isotretinoin. Treatment with the retinoid combination was applied to one side of the face and vehicle was applied to the other, once daily, for 3 months. The percentage of relapses on the side treated with the retinoid combination was almost 17% compared to 43% on the vehicle side; this difference was statistically significant (P=0.021) (Table 1). |
| **Statement 5. Role of Trifarotene in the Management of Acne Sequelae** | | |
| Dual Action in Active Acne and Sequelae | Schleicher et al. (2023) ^4^ | Refer to statement 1: Clinical Trial  This was a 24-week, double-blind, vehicle-controlled, split-face (randomised right face vs left face) study evaluating the efficacy and safety of once-daily trifarotene cream in patients **with moderate-to-severe facial acne and atrophic acne scarring.**  At week 24, a statistically significantly greater reduction in the mean absolute change from baseline in the total atrophic scar count was noted in the trifarotene- vs vehicle-treated area (- 5.9 vs - 2.7; p <0.0001) with differences between sides noted as early as week 2 (- 1.5 vs - 0.7; p = 0.0072). |
|  | Alexis et al. (2024) ^5^ | Refer to statement 1: Clinical Trial  Eligibility criteria were males and females aged 13 to 35, with **moderate facial AV** (Investigator Global Assessment [IGA] score of 3 on the face, ≥20 inflammatory lesions, and ≥25 noninflammatory lesions, excluding nose); **moderate-to-marked AIH (Acne-Induced Hyperpigmentation)** on the face defined as overall disease severity (ODS) hyperpigmentation scale scores of 4–6; and ≤1 AV nodules or cyst (≥1 cm) on the face.  Trifarotene 50 lg/g cream improved significantly from baseline in ODS score versus vehicle (- 1.6 vs. - 1.1, P = 0.03) at Week 12, but scores were comparable between groups at Week 24 (primary endpoint). Trifarotene had a better reduction in PAHPI score at Week 24 (- 18.9% vs. - 11.3% vehicle, P < 0.01). Photography showed improvements in pigmentation and erythema across all skin types. |
| Mechanism of action | Alexis et al. (2024) ^5^ | AIH occurs most often in patients with darker Fitzpatrick skin types (FSTs). AV-induced macular erythema is more frequently observed in those with lighter skin types and is also due to persistent inflammation. Melanocytes can respond to inflammatory stimuli by increasing or decreasing melanin production. In some individuals, inflammation associated with AV induces melanin deposition in the epidermis or dermis, resulting in darker skin at the lesion site.  Trifarotene was designed to be a skin-selective retinoid via stability in the skin and high receptor selectivity for the retinoic acid receptor (RAR) gamma, the predominant RAR subtype expressed in the epidermis.  Trifarotene also has **anti-inflammatory effects, which may dampen melanocyte stimulation and subsequent melanin production**. |
|  | Belmontesi et al. (2023) ^21^ | Controlling inflammation is the key to preventing acne scars. Inflammation plays a crucial role in acne scar formation. The inflammatory reaction in patients with scar-prone acne appears to be stronger, more robust, and more durable. Inflammatory remodelling may alter sebaceous gland structures in scar-prone acne. In the NF-kB-dependent inflammatory pathway involved in acne scar formation, NF-kB activation triggers the release of inflammatory cytokines (ie, TNF-α, IL-1), which in turn stimulate leukocyte diapedesis, causing inflammation and leading to tissue damage and scarring. Prevention of acne scar development requires early, effective control of inflammation.  Trifarotene is the first of the fourth generation of retinoids, specifically designed to have a high selectivity for the RAR-γ receptor. Trifarotene is a potent agonist of RAR, binding specifically to the γ subtype of RAR receptors, which is the predominant receptor type in the skin (~90%).  Trifarotene has 20-fold selectivity for RAR-γ over RAR-α and RAR-β. The RAR-γ receptor is associated with various processes **including cell differentiation and mediation of inflammation**. Trifarotene activates gene expression of retinoid-modulated pathways such as **epidermal differentiation and proliferation.** In-vivo, trifarotene is highly comedolytic. It has shown strong **anti-inflammatory properties and anti-pigmenting properties**. |
|  | Annunziata et al., (2025) ^22^ | Retinoids modulate immune responses and affect dermal remodelling by stimulating the production of procollagen.  The ability of retinoids to modulate collagen synthesis and degradation, among other skin functions (hence their use in the treatment of photoaged skin), is well known. Trifarotene has been shown to modulate molecular pathways involved in extracellular matrix reorganisation and fibrosis, which may partly explain its activity in minimising scar development and abnormal collagen remodelling processes.  In phase 3 and 4 clinical studies, trifarotene, a fourth-generation retinoid with retinoic acid receptor (RAR)-γ selectivity, was found to be well tolerated and effective in treating moderate facial and truncal acne, preventing, and reducing acne-induced scars (AIS) in patients with moderate-to-severe acne and leading to nominal improvement of acne-induced hyperpigmentation (AIH) at week 12 in patients with moderate acne. |
|  | Schleicher et al. (2023) ^4^ | Atrophic acne scarring is speculated to develop from ongoing inflammation in the pilosebaceous unit, since the vast majority of scars originate from papules and pustules and cysts (inflammatory lesions). Inflammatory lesions that resolve slowly are more likely scar, again highlighting the role of ongoing inflammation. Atrophic acne scars are associated with loss of tissue and dermal matrix. Although the pathogenesis of acne scarring is not completely understood, an intricate cell-mediated immune response is central to both active acne lesions and altered wound healing that leads to scarring.  Topical retinoids are well established in active acne treatment, and increasing evidence indicates that these drugs may also improve acne scarring. **Topical retinoids can stimulate dermal fibroblasts to increase procollagen production and can increase epidermal thickness**  Trifarotene, the latest topical retinoid to receive FDA and EU approval, has a unique mechanism of action, binding to retinoic acid nuclear receptor gamma (RAR-c). Furthermore, a transcriptomic and gene expression analysis by Dreno et al. found that trifarotene uniquely affects genes involved in cellular migration, inflammation, and reorganisation of the cellular matrix while downregulating pro-fibrotic macrophages. |
| Combination Modalities | Bhargava et al., (2018) ^23^ | Fractional lasers and radiofrequency offer significant improvement in most types of atrophic acne scars with minimal risks and can be combined with all other treatment options. Combination therapies typically provide superior outcomes than solo treatments. |
|  | Kashetsky et al., (2024) ^24^ | This concept is supported by several high-quality studies which have also found that combination therapies have superior efficacy rates as compared to monotherapy in hyperpigmentation disorders. For example, a systematic review by Mukovozov et al. evaluating treatment modalities for lentigines and their corresponding clinical outcomes found that combination therapies had greater efficacy rates as compared to laser, topical, cryotherapy and peel monotherapies. |
|  | Kravvas & Al-Niaimi, (2017) ^25^ | The most impressive results were seen following application of chemical peels, with excellent response rates often achieved in >70% of patients. Combination treatments are shown to have a favourable outcome too. When cryo-roller treatment was used in addition to subcision, response rates went up by 17%, compared with subcision used in isolation. In addition, particularly effective seem to be the combination treatments with microneedling/GA peeling and microneedling/PRP. |
|  | Belmontesi et al., (2023) ^21^ | The results observed in this case series show that the sequential treatment with topical trifarotene and injectable NASHA gel as skin booster can be effective in the progressive reduction of acne scarring, potentially related to a synergic effect of skin remodelling and collagen stimulation. |
| Potential Benefit in Acne-Induced Erythema | Alexis et. al (2024) ^5^ | As can be seen in the patient images in Figure 3a, b, marked **improvement occurred in focal facial erythema** **in light-skinned individuals**, which may be similarly referred to as acne-induced erythema, even though this study was not designed to assess erythema. **The results show improvements in both AIH and erythema**, which are promising for long-term treatment and management of AV and its associated sequelae. |
| Scar Management | Belmontesi et al. (2023) ^21^ | NASHA gel skin booster administered in **three sessions at 1-month intervals** was shown to improve skin elasticity, reduce roughness, improve skin tone, improve skin hydration, reduce fine lines, and **reduce the appearance of atrophic acne scar.** |
| **Statement 6. Management of Side Effects** | | |
| Local Tolerability | Schleicher et al. (2023) ^4^ | Both trifarotene and its vehicle were well tolerated with treatment emergent adverse events (TEAEs) occurring in 5.8% of trifarotene-treated sides of the face and 2.5% of vehicle-treated sides. The most common adverse events were local events, such as skin tightness, pruritus, erythema, and rash. |
|  | Tan et al. (2019) ^1^ | Refer to Statement 3: Addressing Skin Barrier Needs |
|  | Blume-Pytavi et al. (2020) ^2^ | Refer to Statement 3: Addressing Skin Barrier Needs  Local tolerability on the trunk was better than on the face. A possible explanation could be that the epidermis of the back is thicker than of the face. Facial and truncal skin are exposed to different environmental challenges. Facial skin is more frequently exposed to environmental sources of irritation and damage, and often remains unprotected against external triggers such as UV light, temperature and pollution, while the skin on the trunk is more frequently exposed to heat, moisture and occlusion. |
| Common AEs | Tan et al. (2019) ^1^ | Refer to Statement 3: Addressing Skin Barrier Needs |
|  | Blume-Pytavi et al. (2020) ^2^ | Refer to Statement 3: Addressing Skin Barrier Needs |
|  | Trifarotene - US Product information. (2022) ^26^ | Commonly reported adverse reactions in the two 12-week phase 3 clinical trials included irritation (n=91, 7.5%), pruritus (n=29, 2.4%), and sunburn (n=32, 2.6%) among a total of 1220 patients with acne vulgaris of the face and trunk receiving trifarotene treatment. |
| Transient Nature | Blume-Pytavi et al. (2020) ^2^ | Refer to Statement 3: Addressing Skin Barrier Needs  As observed with other retinoids, the majority **of these local adverse events occurred during the first weeks of treatment, subsiding thereafter; all were mild or moderate in intensity.** |
| Photoprotection | Goh et al. (2023) ^27^ | UV radiation can also increase the thickness of the stratum corneum and cause microbial dysbiosis and thus aggravate acne flares.  Furthermore, **some topical and systemic acne therapies can increase the risks of phototoxicity.** Physicians should therefore educate patients on avoidance of midday sun and use of protective clothing as the first essential steps for photoprotection along with use of sunscreens. |
|  | Goh et al. (2024) ^28^ | Table 5 provides an example of cleansing, treatment, moisturisation, and photoprotection plan for four skin conditions. For adolescent acne, protect with sunscreen with UVA and UVB SPF 30 was recommended, in addition to cleansing treatment, and moisturising routines. |
| Optimum Use of Cleansers and Moisturisers | Goh et al. (2024) ^28^ | Optimum use of cleansers and moisturisers in combination with prescription therapy for acne can **prevent the appearance of new lesions, reduce inflammation, improve skin barrier and boost tolerability and adherence to topical therapy**. Reduction in treatment related side effects and need for topical antibiotics in acne are other significant advantages. |
| Acne Flare-ups after retinoid irritation | Schlessinger et. al (2007) ^29^ | Results from the three Phase 3 clinical trials demonstrate that treatment with a 0.025% formulation of tretinoin used as monotherapy provoked acne flaring in a subset of subjects. Almost twice as many study participants with mild acne at baseline demonstrated a 10 percent or greater increase in number of inflammatory lesions as compared to subjects in the vehicle arm (15.4% vs 8.7%). However, this effect was seen only for those with mild acne at baseline and not in those with moderate or severe baseline inflammatory acne. There was no evidence that clindamycin phosphate 1.2%/tretinoin 0.025% gel provoked acne flaring. |
|  | Leyden et. al (2012) ^30^ | We also demonstrated that topical retinoid therapy is not associated with an increase in inflammatory lesions following the first week of treatment regardless of using the VLS model of percentage-based definitions. |
|  | [Yentzer](https://pubmed.ncbi.nlm.nih.gov/?term=Yentzer+BA&cauthor_id=19746671)  et al. (2009) ^31^ | This review aimed to review the available data from clinical trials for evidence of initial worsening of acne with topical retinoids. No primary data from clinical trials were identified to support the dogma of acne worsening secondary to topical retinoids. Available data point to topical retinoids improving acne, even during the first couple weeks of treatment. |
| Cleanse-treat-moisturise-photoprotect (CTMP®) Routine | Goh et al. (2024) ^28^ | A holistic skincare routine (cleansing, treatment, moisturisation, and photoprotection [CTMP]) was recommended for four skin types, including those with acne. This routine included cleansing 2 times a day to remove dirt and sebum, treatment using trifarotene, moisturising 2 times a day to counter dryness from retinoids, and protecting with subscene UVA + UVB SPF 30. |
| Special Populations | See et al. (2024) ^32^ | Additionally, Asian skin is reported to have lower trans-epidermal water loss (TEWL), higher water content, and greater sweat gland activity than other skin types, meaning that **Asian skin is likely to be more susceptible to irritation from chemical stimuli.** |
| Patient Alerts | Eichenfield et al. (2022) ^33^ | In addition, patients should be educated to **avoid applying trifarotene to cuts, abrasions, or eczematous or sunburned skin.** |
| **Statement 7. Use in Special Populations** | | |
| Adolescent & Adult Populations | Trifarotene - US Product Information (2022) ^26^ | AKLIEF Cream is a retinoid indicated for the topical treatment of acne vulgaris in **patients 9 years of age and older.** |
|  | Trifarotene – Malaysia Product Information. (2022) ^11^ | AKLIEF is indicated for the topical treatment of Acne Vulgaris of the face and/or the trunk in **patients from 12 years of age and older**, when many comedones, papules and/or pustules are present. |
|  | Tan et. al (2019) ^1^ | The eligibility criteria were patient **age 9 years and older.** |
|  | Blume-Peyvati (2020) ^2^ | The study included **patients 9 years of age or older** at enrolment. |
| Darker Skin Types | Schleicher et al. (2023) ^4^ | A total of 121 patients participated, with a mean age of 22 years. Most (84.3%) were 18 years or older, female (72.7%), and white (80.2**%), 30.6% had darker skin types (Fitzpatrick IV and V)**, and 21.5% self-identified as Hispanic. |
|  | Alexis et al. (2024) ^5^ | A total of 60 patients were included in the trifarotene group and 63 in the vehicle group, among which 22 (36.7%) and **23 (36.5%) had darker skin types (Fitzpatrick IV and V)** in the trifarotene and vehicle groups respectively. |
| Pregnancy / Lactation | Wagner et al. (2020) ^10^ | Clinical pharmacology data demonstrate that trifarotene 50 µg/g cream, the to-be-marketed formulation, **generates low systemic absorption when applied daily under maximal use conditions.** With a trifarotene daily dose of 2 g, the mean Cmax in adults had a range of <5 to 8 pg/mL and in children a range of <5 to 9 pg/mL. In the TQT study of healthy subjects, 12 g once daily on 6000 cm2 resulted in a mean Cmax of 33 ± 34 pg/mL (<5-187 pg/mL). |
|  | Trifarotene-Malaysia Product Information (2022) ^11^ | Aklief is contraindicated (see section 4.3) during pregnancy or in women planning a pregnancy. **If the product is used during pregnancy, or if the patient becomes pregnant while taking this drug, treatment should be discontinued.** |
| Exclusions | Reynolds et. al (2024) ^13^ | According to the clinical management of acne vulgaris pathway (Fig 1), nodulocystic acne is typically treated with systemic therapies. |
|  | Thiboutot et al. (2027) ^14^ | As suggested in this paper, nodular and/or conglobate acne can be treated using oral isotretinoin or fixed combination plus oral antibiotics. |
| Use in 9 – 11 year olds | Wagner et al. (2020) ^10^ | The clinical pharmacology of topical trifarotene up to 100 µg/g was extensively investigated through 2 maximal usage pharmacokinetic trials (MUsT) conducted in adult (≥18 years) and paediatric patients (9‐17 years) with moderate-to-severe acne and two studies conducted in healthy volunteers: 1 thorough QTC study and 1 drug‐drug interaction study with concomitantly administered oral levonorgestrel (0.15 mg)/ethinyl estradiol (0.03 mg). In MUsT 1, patients were aged 18 to 34 years, with an acne severity grade of 4 (severe acne) on the 0 to 4 grade Investigator's Global Assessment (IGA, graded from 0 = none to 4 = severe), and at least 30 noninflammatory and 40 inflammatory lesions on the face at Screening and Baseline. In MUsT 2, participants were aged 10 to 17 years; subjects up to 11 years were required to have an IGA ≥3 (moderate-to-severe acne), while patients aged 12 to 17 years had to have an IGA = 4 and at least 40 noninflammatory and 25 inflammatory lesions on the face. Once‐daily applications of trifarotene 50‐ or 100‐µg/g cream for 29 days were performed by a qualified person.  Systemic absorption of trifarotene was generally unquantifiable in the target population, especially when applied at 50 μg/g.  **MUsT 1: Adults**  After 29 applications of trifarotene 50 μg/g cream, trifarotene plasma concentrations were quantifiable in 7 of 19 (37%) patients, with a Cmax ranging from 5 to 10 pg/mL and an AUC0-24h from 75 to 104 pg • h/mL. Tmax averaged approximately 4 hours in both the 50- and 100-μg/g groups.  **MUsT 2: Paediatric**  After 29 applications of trifarotene 50 μg/g cream under maximal use conditions, trifarotene plasma concentrations were quantifiable in 3 of 17 (18%) patients aged 12 to 17 years, with Cmax ranging from 7 to 9 pg/mL and AUC0-24h from 89 to 106 pg • h/mL. A peak plasma concentration (tmax) was observed approximately 4 hours after application for both groups.  Quantifiable data obtained with trifarotene 100-μg/g cream confirmed the absence of a sex or age effect on  the systemic exposure to trifarotene. |
|  | Tan et. al (2019) ^1^ | Refer to Statement 1: Adolescent & Adult Populations |
|  | Blume-Peyvati (2020) ^2^ | Relatively few patients aged between 9 and 11 years were enrolled in the study (n=18, 4.0%). This was due the low prevalence with truncal acne found in this group. |
| **8. Patient Education and Adherence** | | |
| Adherence Imperative | Alexis et al. (2024) ^5^ | As shown in Figure 4, >90% of subjects in the trifarotene group agreed that the cleanser and the moisturising lotion helped reduce skin irritation and dryness and increase compliance with their acne treatment. |
|  | Tan et. al (2019) ^1^ | Patients were also instructed to cleanse the skin and not apply moisturiser 1 hour before or 1 hour after application of the study drug. Use of moisturiser was encouraged from the initiation of treatment. **The implementation of routine standard skin care**, such as use of noncomedogenic moisturisers and gentle cleanser, and dosing regimen adjustments were **sufficient to ensure treatment management and compliance in the majority of patients**. |
| Psychosocial Factors | Tan et. al. (2021) ^34^ | **Patient satisfaction with treatment is one of the most important aspects to consider in ongoing management to improve adherence to treatment regimens**; however, this is multifactorial and includes whether patients are satisfied with **the improvement in their acne with the treatment regimen** and whether they are satisfied with **their appearance**. Currently, the majority of treatment algorithms in national and regional clinical management guidelines do not incorporate patient-oriented treatment goals or patient satisfaction as an outcome. Thus, the PACE panel has recommended addressing patient satisfaction in consultations to improve adherence and ultimately improve patient outcomes. |
|  | Callender et al. (2022) ^35^ | Education and training for clinicians and researchers about acne and PIH in patients with SOC may improve diagnoses and treatment outcomes; **education for patients about their dermatologic conditions, medications, and skin care may help to manage expectations and improve treatment adherence.** |
| Support Materials | Myhill et al (2017) ^36^ | Subjects with acne were randomised to receive once-daily A/BPO for 12 weeks plus (1) supplementary educational material (SEM) in addition to SOCPE (standard-of-care patient education [SOCPE], including package insert and oral instruction); (2) SOCPE only with two additional visits; or (3) SOCPE only. The SEM included a 3-min video about the mode of action of A/BPO, the method of application, treatment expectations, and management of irritation. Subjects also received an information card containing the key messages of the video, as well as additional information about acne and A/BPO available online. The A/BPO + SEM group had more subjects with greater than 75% adherence (45%, 30.4%, and 25%, respectively). According to the subject appreciation questionnaire, the SEM was more helpful to adhere to treatment (56.7%) versus more visits (32.3%) and A/BPO alone (15.2%), better use the product (70%, 61.3%, and 54.5%, respectively), and better manage skin irritation (53.3%, 48.4%, and 36.4%, respectively). |
|  | Ling et al. (2023) ^37^ | This study aims to determine the effectiveness of medical education and counselling on treatment adherence and disease severity. Patients in the intervention group received a 10 min video presentation on acne, followed by treatment counselling. The video answered the ten most frequently asked questions regarding acne. During the counselling, the pharmacist would demonstrate the medication administration methods, explaining the possible side effects and their management. Patients were **reassured of the importance of treatment adherence even though they noticed no immediate progress**. In addition, an acne information leaflet was distributed to them to consolidate their understanding of acne. With intervention, patients have better adherence to topical medication (5% benzoyl peroxide gel: 71% vs 57.9%, p= 0.031; 0.05% tretinoin cream: 58.7% vs 45.4%, p= 0.044) at week 12. Overall, with intervention, a significantly higher percentage of improvement in disease severity was noted (47.3% vs. 39.1%, p=0.044). |
| Brief-contact | Annunziata et. al (2025) ^22^ | Practical suggestions to minimise retinoid-induced skin irritation are listed in Table 2, including short-contact therapy (washing the face ≤30 min after application). |
| Skincare regimen | Goh et al. (2023) ^27^ | A dermatologist-guided holistic skin care routine is essential for patients with acne, rosacea, atopic dermatitis, and idiopathic sensitive skin syndrome to improve patient confidence and reduce confusion over product selection. The consensus recommendations presented here highlight the importance of cleansing, moisturisation, and photoprotection in holistic skin care and how it can be utilised as a communication tool for physicians and patients to achieve overall better patient compliance, satisfaction, and treatment outcomes.  Moisturisers are crucial in improving treatment adherence by mitigating skin dryness and irritation, which are the common side effects of acne therapy. In mild–to-moderate acne, a regimen of cleanser and an active formulation moisturiser reduced the mean total lesion count (6.9% vs. 1.4%), pustular lesions (*p* < 0.05), and sebum levels (*p* < 0.01) and reduced colonisation of *Propionibacterium acne* (49.4% vs. 3.2%) compared to vehicle.  The panel advises that moisturisers for acne should improve stratum corneum water content, should not affect the efficacy of topical acne treatment, and should improve tolerance to topical treatment. Additionally, hypoallergenic, alcohol-free, and water-based moisturisers are recommended. |
|  | Goh et al. (2024) ^28^ | **Optimum use of cleansers and moisturisers in combination with prescription therapy for acne** can prevent the appearance of new lesions, reduce inflammation, improve skin barrier and **boost tolerability and adherence to topical therapy.** Reduction in treatment related side effects and need for topical antibiotics in acne are other significant advantages. Cleansers and moisturisers are also beneficial in rosacea by reducing facial dryness, burning, and stinging, and improving tolerability to topical medication. Similarly, moisturisers form the cornerstone of AD treatment by strengthening the skin barrier function, decreasing stratum corneum moisture loss, and reducing the number and frequency of flares. Therapeutic moisturisers for AD offer additional anti-inflammatory, antipruritic, and antioxidant benefits, while some others demonstrate the steroid-sparing effect.  Ultraviolet radiation is known to affect skin barrier function, trigger skin inflammation and aggravate atopic dermatitis, rosacea, and acne flares. Some medications can increase the risk of phototoxicity, and hence, photoprotection is recommended with topical retinoids, oral tetracyclines, and benzoyl peroxide-containing products. |
| Layering Techniques | Trifarotene – Malaysia Product Information (2022) ^11^ | The use of a moisturiser is recommended as needed from the initiation of treatment, while  allowing sufficient time before and after the application of Aklief cream to allow the skin to dry. |
|  | Parsa (2025) ^38^ | In this study, researchers tested retinol 0.1% cream and tretinoin 0.025% cream in combination with lightweight water gel and water cream moisturisers on human skin biopsies from the abdominoplasty of a Caucasian woman. Human skin explants were treated topically for 48 hours with 1) the retinoid formula alone; 2) a 2-step regimen of retinoid plus moisturiser or moisturiser plus retinoid (“open sandwich); or 3) a 3-step regimen of moisturiser plus retinoid plus moisturiser (“full sandwich”). Results from the study showed that explants treated with the “full sandwich” method had reduced bioactivity (p<0.05), demonstrated by the HBEGF gene expression. Explants treated with the “open sandwich” regimen in either order of application maintained comparable bioactivity to the retinoid treatment alone, as shown by *HBEGF* gene expression. The study suggested that a 3-step regimen of moisturiser plus retinoid plus moisturiser (“full sandwich”) **reduces retinoid bioavailability by 3-fold** compared to a 2-step regimen of retinoid plus moisturiser or moisturiser plus retinoid (“open sandwich), likely due to dilutional and penetration effects. |
| **Statement 9. Maintenance Therapy** | | |
| Efficacy | Blume-Peyvati (2020) ^2^ | Refer to Statement 1: Clinical Trial |
|  | Schleicher et al. (2023) ^4^ | Refer to Statement 1: Clinical Trial |
| Safety and tolerability | Blume-Peyvati (2020) ^2^ | Refer to Statement 3: Addressing Skin Barrier Needs |
|  | Schleicher et al. (2023) ^4^ | Both trifarotene and its vehicle were well tolerated with TEAEs occurring in 5.8% of trifarotene-treated sides of the face and 2.5% of vehicle-treated sides. A summary of adverse events is shown in Table 3. The most common adverse events were local events, such as skin tightness, pruritus, erythema, and rash. All events were mild to  moderate in severity. |
| **Statement 10. Addressing Unmet Needs & Future Directions** | | |
| Truncal Acne | Tan et al. (2022) ^39^ | Truncal acne (inclusive of the anterior chest, upper back, shoulders) is **frequently underdiagnosed** despite affecting around half of individuals that have facial acne. In consultations, patients may focus primarily on facial acne, since it is readily visible to others, highlighting the importance of performing a physical exam of the face, chest and back to evaluate severity and extent of disease. In a referral cohort of 965 patients with acne, patient reporting was consistent with clinical evaluation in 92 percent of cases for facial acne but only around 70 percent for truncal acne. In a community-based US study, 22 percent of the patients with facial acne as the primary reason for consultation did not spontaneously mention truncal involvement during the initial consultation but it was detected during the physical examination. |
|  | Woo YR and Kim HS (2022) ^40^ | The prevalence of truncal acne has not yet been well established to date. **Previous studies report that about 48–52% of facial acne patients also have truncal acne**. In 2007, Del Rosso et al. examined 696 patients aged 14 to 20 years with acne and found that 52.3% exhibited truncal involvement. Among them, 10.6% showed truncal acne scarring. Isaacsson et al. reported that 50% of Brazilian adolescents had acne on their chest or back. A large-scale international study of 2926 adult females found that 48.8% of the patients with facial acne also had truncal acne. Recently, Dreno et al. reported that a family history of acne was associated with the extension of acne to the trunk. |
|  | Ko et al. (2024) ^41^ | Since most studies on acne have focused on the face, **data on clinical presentations and management for truncal acne are insufficient in the literature.**  The recommendations serve as a foundation for developing guidelines and can aid in improving care for truncal acne patients by increasing attention during consultations. |
|  | Schleicher et al. (2023) ^4^ | **Multidisciplinary research of truncal acne is needed** to understand its pathogenic characteristics and **proper management options**. |
|  | Tan et. al (2019) ^1^ | Because the pathophysiology and clinical presentations of facial and truncal acne are considered to be similar, clinicians often apply the same therapeutic approach for facial and nonfacial lesions despite a lack of evidence in truncal AV.3-5 A variety of treatment options are currently available for AV, but **they have not been rigorously studied in truncal disease.** |
|  | Blume-Peyvati (2020) ^2^ | Even though many topical and systemic treatments have been used in the past to treat acne, **none has been specifically developed and clinically studied for the treatment of both facial and truncal acne**. |
|  | See et. al (2024) ^32^ | To further improve the standard of care for patients with truncal acne, **we suggest that specific evidence is needed to support the efficacy of established acne therapies for the treatment of acne on the trunk**, which can then be used to substantiate future guideline updates in the Asia-Pacific region |
| Fast Improvement in Atrophic Scars & Hyperpigmentation | Schleicher et al. (2023) ^4^ | Refer to statement 1: Clinical trial.  A **significantly superior reduction in atrophic scar count occurred on trifarotene-treated sides** **of the face** compared to vehicle-treated sides. At week 24, the mean absolute change from baseline was − 6.2 ± 5.6 on the trifarotene-treated area compared to − 2.8 ± 3.9 on the vehicle area (Δ − 3.2, P < 0.0001). This was primarily due to a greater amount of reduction in 2–4 mm atrophic acne scars. As shown, **there was a difference between treated sides as early as week 2** (P = 0.001), which continued at week 4 (P = 0.007) and weeks 8 through 20 (P < 0.0001). The total scar counts were similar between groups at baseline (Table 1), but **by week 24, the total scar count was 5.4 ± 5.6 for trifarotene-treated areas and 9.1 ± 8.6 for vehicle-treated areas (percent reductions of 55.2% and 29.9%, respectively)**. |
|  | Alexis et al. (2024) ^5^ | Refer to statement 1: Clinical trial.  Trifarotene had **a better reduction in PAHPI score at Week 24** (−18.9% vs. −11.3% vehicle, P < 0.01). **Lesion count reductions were higher with trifarotene at Week 12 versus vehicle** (P < 0.001) and at **Week 24** (P < 0.05), as **were IGA success rates versus vehicle at Weeks 12** (P < 0.05) **and 24** (P < 0.05). |

**Abbreviations:** AIH, acne-induced hyperpigmentation; AIS, acne-induced scarring; AQP3, aquaporin 3; ATP11B, ATPase phospholipid transporting 11B; AV, acne vulgaris; BTB, broad-complex, tramtrack, and bric-a-brac domain; CAAX, C-terminal motif involved in prenylation; CASS, comprehensive acne severity scale; CHL1, cell adhesion molecule L1 like; CRABP2, cellular retinoic acid binding protein 2; CXCR2, C-X-C motif chemokine receptor 2; CYP26A1, cytochrome P450 family 26 subfamily A member 1; DHRS9, dehydrogenase/reductase 9; DNA, deoxyribonucleic acid; DST, dystonin; EF, elongation factor; EFHD2, EF-hand domain family member D2; ELF3, E74 like ETS transcription factor 3; ETS, E26 transformation-specific sequence; F3, coagulation factor III; FGFR2, fibroblast growth factor receptor 2; FOSL1, FOS like 1; GPX2, glutathione peroxidase 2; HBEGF, Heparin-binding EGF-like growth factor; ID1, inhibitor of DNA binding 1; IGA, investigator global assessment; IL-1, interleukin 1; IL1RN, interleukin 1 receptor antagonist; KLK6, kallikrein related peptidase 6; KLK8, kallikrein related peptidase 8; KLK10, kallikrein related peptidase 10; KRT4, keratin 4; MME, membrane metalloendopeptidase; NF-kB, nuclear factor kappa B; PAHPI, post-inflammatory hyperpigmentation index; PADI1, peptidyl arginine deiminase 1; PGA, physician global assessment; PPARD, peroxisome proliferator-activated receptor delta; PRRG4, proline rich and Gla domain 4; PRSS27, serine protease 27; P2RY2, purinergic receptor P2Y2; RAR-γ, retinoic acid receptor gamma; RARc, retinoic acid receptor gamma; RHCG, Rh family C glycoprotein; RIT1, Ras like without CAAX 1; S100A9, S100 calcium binding protein A9; SERPINA12, serpin family A member 12; SPF, sun protection factor; SPON1, spondin 1; STRA6, stimulated by retinoic acid 6; T+D: trifarotene cream plus doxycycline; TEAE, treatment-emergent adverse event; TEWL, transepidermal water loss; TNF-α, tumor necrosis factor alpha; TQT, thorough QT; TYMS, thymidylate synthetase; UVA, ultraviolet A; UVB, ultraviolet B; V+P: trifarotene vehicle plus doxycycline placebo; ZBTB20, zinc finger and BTB domain containing 20.

***** The evidence included in this table was directly extracted from the cited articles without any paraphrasing. A p value less than 0.05 is considered statistically significant.

## Table A4. Pre-Advisory Meeting Survey, Agreement Rating and Feedback / Comment on Clinical Advisory Statement

| **Statement No.** | **Rating Outcome** | **Feedback / Comment** |
| --- | --- | --- |
| **Statement 1: Integrate Trifarotene into Existing Acne Management Algorithms** |  | First line for patients with both facial and truncal acne. |
|  |  | For facial acne not responding well to Differin or for better maintenance post-isotretinoin. |
| **Statement 2: Patient Selection Criteria** |  | Nodulocystic acne is not a contraindication, just because it may not be good enough. |
| **Statement 3: Dosage and Administration** |  | Suggest to start slow and build up. |
|  |  | It would be helpful to state the extent beyond visible lesions. 1cm beyond active acne? |
| **Statement 4: Combination Therapy (Other topical agents & Systemic Agents for acne)** |  | Can consider combination with isotretinoin in severe acne. |
| **Statement 5: Role of Trifarotene in the Management of Acne Sequelae** |  | Suggest removing the word "primary" from this statement as there are other topical agent which can reduce sequelae of acne especially hyperpigmentation. Suggest changing to: "Trifarotene is recommended as an option of topical therapy for managing acne sequelae in patients with acne induced scarring and acne induced macular hyperpigmentation." |
| **Statement 6: Management of Side Effects** |  | Start slow and build up can mitigate side effects. |
| **Statement 7: Use in Special Populations** |  | I am uncertain if it is appropriate to describe the safety data as "robust" for adolescents aged 9–11 years, given that the study by Blume-Peytavi et al. (2020) included only 4% of participants within this age group. Although the PERFECT 1 and 2 trials mentioned including individuals aged 9 and above, the data was categorised broadly as <18 and >18 years, which limits age-specific conclusions. |
|  |  | Suggest changing the age group to ≥12 as this is the age group approved for Trifarotene in Malaysia. Suggest the following: " Trifarotene is supported by robust safety data in adolescents (≥ 12 years) and individuals with darker skin phototypes, who may benefit from reduced risk of post-inflammatory hyperpigmentation. |
| **Statement 8: Patient Education and Adherence** |  | Is there any other users experiencing purging post utilisation of trifarotene? |
| **Statement 9: Maintenance Therapy** |  | N/A |
| **Statement 10: Addressing Unmet Needs & Future Directions** |  | Steroid-induced acne. |
|  |  | The need to properly define which patients should be prescribed adapalene and which to be given trifarotene. Objective comparisons between the 2 groups in terms of efficacy and safety is needed. |

## References

1 J. Tan, D. Thiboutot, G. Popp *et al.* Randomized phase 3 evaluation of trifarotene 50 mug/g cream treatment of moderate facial and truncal acne. *J Am Acad Dermatol* 2019; 80: 1691-1699.

2 U. Blume-Peytavi, J. Fowler, L. Kemeny *et al.* Long-term safety and efficacy of trifarotene 50 mug/g cream, a first-in-class RAR-gamma selective topical retinoid, in patients with moderate facial and truncal acne. *J Eur Acad Dermatol Venereol* 2020; 34: 166-173.

3 J. Q. Del Rosso, S. M. Johnson, T. Schlesinger *et al.* A Randomized, Controlled Trial of Trifarotene Plus Doxycycline for Severe Acne Vulgaris. *J Clin Aesthet Dermatol* 2022; 15: E53-E59.

4 S. Schleicher, A. Moore, E. Rafal *et al.* Trifarotene Reduces Risk for Atrophic Acne Scars: Results from A Phase 4 Controlled Study. *Dermatol Ther (Heidelb)* 2023; 13: 3085-3096.

5 A. Alexis, J. Q. Del Rosso, S. Forman *et al.* Importance of treating acne sequelae in skin of color: 6-month phase IV study of trifarotene with an appropriate skincare routine including UV protection in acne-induced post-inflammatory hyperpigmentation. *Int J Dermatol* 2024; 63: 806-815.

6 J. Aubert, D. Piwnica, B. Bertino *et al.* Nonclinical and human pharmacology of the potent and selective topical retinoic acid receptor-gamma agonist trifarotene. *Br J Dermatol* 2018; 179: 442-456.

7 E. Thoreau, J.-M. Arlabosse, C. Bouix-Peter *et al.* Structure-based design of Trifarotene (CD5789), a potent and selective RARγ agonist for the treatment of acne. *Bioorganic & Medicinal Chemistry Letters* 2018; 28: 1736-1741.

8 B. Dreno, R. Chavda, V. Julia, A. Khammari, S. Blanchet-Rethore, J. K. Krishnaswamy. Transcriptomics Analysis Indicates Trifarotene Reverses Acne-Related Gene Expression Changes. *Front Med (Lausanne)* 2021; 8: 745822.

9 M. Shergill, M. U. Ali, M. Abu-Hilal. Comparison of the Efficacy of Clascoterone, Trifarotene, and Tazarotene for the Treatment of Acne: A Systematic Literature Review and Meta-Analysis. *Dermatol Ther (Heidelb)* 2024; 14: 1093-1102.

10 N. Wagner, K. Benkali, A. Alio Saenz, M. Poncet, M. Graeber. Clinical Pharmacology and Safety of Trifarotene, a First-in-Class RARgamma-Selective Topical Retinoid. *J Clin Pharmacol* 2020; 60: 660-668.

11 Trifarotene - Malaysia Product Information. In. <https://quest3plus.bpfk.gov.my/front-end/attachment/111542/pharma/555110/555110_20221220_211847_.pdf>: National Pharmaceutical Regulatory Agency (NPRA) [Bahagian Regulatori Farmasi Negara], 2024.

12 Ministry of Health Malaysia Clinical practice guidelines: management of acne vulgaris 2nd edition. In. Malaysia: Malaysian Health Technology Assessment Section (MaHTAS), Medical Development Division, Ministry of Health Malaysia, 2022.

13 R. V. Reynolds, H. Yeung, C. E. Cheng *et al.* Guidelines of care for the management of acne vulgaris. *J Am Acad Dermatol* 2024; 90: 1006.e1001-1006.e1030.

14 D. Thiboutot, B. Dréno, A. Abanmi *et al.* Practical management of acne for clinicians: An international consensus from the Global Alliance to Improve Outcomes in Acne. *Journal of the American Academy of Dermatology* 2017; 78.

15 S. M. Johnson, R. Chavda, J. C. DuBois. Subject Satisfaction with Trifarotene 50 mug/g Cream in the Treatment of Facial and Truncal Acne Vulgaris: A Case Series. *Dermatol Ther (Heidelb)* 2020; 10: 1165-1173.

16 B. Martin, C. Meunier, D. Montels, O. Watts. Chemical stability of adapalene and tretinoin when combined with benzoyl peroxide in presence and in absence of visible light and ultraviolet radiation. *British Journal of Dermatology* 1998; 139: 8-11.

17 H. Grobel, S. A. Murphy Chapter 77 - Acne Vulgaris and Acne Rosacea. In: *Integrative Medicine (Fourth Edition)*. Elsevier, 2018, 759-770.e755.

18 S. M. Miranti. Maintenance Acne Treatment With Topical Tazarotene after Oral Isotretinoin: Overview and Case Reports. *The Journal of Clinical and Aesthetic Dermatology* 2024; 17: S14.

19 R. Vender, R. Vender. Double‐Blinded, Vehicle‐Controlled Proof of Concept Study to Investigate the Recurrence of Inflammatory and Noninflammatory Acne Lesions Using Tretinoin Gel (Microsphere) 0.04% in Male Patients after Oral Isotretinoin Use. *Dermatology Research and Practice* 2012; 2012: 736532.

20 M. Truchuelo, N. Jiménez, D. Mavura, P. Jaén. Assessment of the efficacy and safety of a combination of 2 topical retinoids (RetinSphere) in maintaining post-treatment response of acne to oral isotretinoin. *Actas Dermo-Sifiliográficas* 2015; 106: 126-132.

21 M. Belmontesi. Sequential Treatment With Topical Trifarotene and Injectable NASHA Gel in Acne Scars: A Case Series. *J Drugs Dermatol* 2023; 22: 502-506.

22 M. C. Annunziata, M. Barbareschi, V. Bettoli *et al.* A Real-World Approach to Trifarotene Treatment in Patients with Acne and Acne Sequelae Based on the Experience of the Italian Acne Board. *Dermatol Ther (Heidelb)* 2025.

23 S. Bhargava, P. R. Cunha, J. Lee, G. Kroumpouzos. Acne Scarring Management: Systematic Review and Evaluation of the Evidence. *Am J Clin Dermatol* 2018; 19: 459-477.

24 N. Kashetsky, A. Feschuk, M. E. Pratt. Post-inflammatory hyperpigmentation: A systematic review of treatment outcomes. *J Eur Acad Dermatol Venereol* 2024; 38: 470-479.

25 G. Kravvas, F. Al-Niaimi. A systematic review of treatments for acne scarring. Part 1: Non-energy-based techniques. *Scars Burn Heal* 2017; 3: 2059513117695312.

26 Trifarotene - US Product Information. In. <https://www.accessdata.fda.gov/drugsatfda_docs/label/2022/211527Orig1s001lbl.pdf>: Drugs@FDA: FDA-Approved Drugs, 2022.

27 C. L. Goh, Y. Wu, B. Welsh *et al.* Expert consensus on holistic skin care routine: Focus on acne, rosacea, atopic dermatitis, and sensitive skin syndrome. *J Cosmet Dermatol* 2023; 22: 45-54.

28 C. L. Goh, Y. Wu, B. Welsh *et al.* Challenges and real-world solutions for adoption of holistic skincare routine (cleansing, treatment, moisturization, and photoprotection) in acne, rosacea, atopic dermatitis, and sensitive skin: An expert consensus. *J Cosmet Dermatol* 2024; 23: 2516-2523.

29 J. Schlessinger, A. Menter, M. Gold *et al.* Clinical safety and efficacy studies of a novel formulation combining 1.2% clindamycin phosphate and 0.025% tretinoin for the treatment of acne vulgaris. *Journal of drugs in dermatology: JDD* 2007; 6: 607-615.

30 J. J. Leyden, N. Preston, L. A. Johnson, R. Gottschalk. Effects of topical retinoid therapy on acne lesions: a psychometric assessment. *Cutis* 2012; 90: 46-50.

31 B. A. Yentzer, R. W. McClain, S. R. Feldman. Do topical retinoids cause acne to" flare"? *Journal of drugs in dermatology: JDD* 2009; 8: 799-801.

32 J. A. See, R. Chavda, K. M. Kon *et al.* A review of the topical management of acne and its associated sequelae in the Asia-Pacific region with a spotlight on trifarotene. *Int J Dermatol* 2024; 63: 704-713.

33 L. Eichenfield, P. Kwong, S. Lee, D. Krowchuk, K. Arekapudi, A. Hebert. Advances in Topical Management of Adolescent Facial and Truncal Acne: A Phase 3 Pooled Analysis of Safety and Efficacy of Trifarotene 0.005% Cream. *J Drugs Dermatol* 2022; 21: 582-586.

34 J. Tan, A. Alexis, H. Baldwin *et al.* The Personalised Acne Care Pathway—Recommendations to guide longitudinal management from the Personalising Acne: Consensus of Experts. *JAAD International* 2021; 5: 101-111.

35 V. D. Callender, H. Baldwin, F. E. Cook-Bolden, A. F. Alexis, L. Stein Gold, E. Guenin. Effects of Topical Retinoids on Acne and Post-inflammatory Hyperpigmentation in Patients with Skin of Color: A Clinical Review and Implications for Practice. *Am J Clin Dermatol* 2022; 23: 69-81.

36 T. Myhill, W. Coulson, P. Nixon, S. Royal, T. McCormack, N. Kerrouche. Use of Supplementary Patient Education Material Increases Treatment Adherence and Satisfaction Among Acne Patients Receiving Adapalene 0.1%/Benzoyl Peroxide 2.5% Gel in Primary Care Clinics: A Multicenter, Randomized, Controlled Clinical Study. *Dermatology and Therapy* 2017; 7: 515-524.

37 W. Y. Ling, C. H. Loo, M. A. Nurul Shafaril Niza, J. L. Tan, M. A. Norazlima, W. C. Tan. The effect of medical education and counselling on treatment adherence and disease severity in patients with acne vulgaris: a non-randomised interventional study. *Med J Malaysia* 2023; 78: 263-269.

38 R. Parsa, W. Li, R. Patel Tretinoin and retinol bioactivity are retained when layered with adjunctive water gel moisturizer or a water cream moisturizer in an “open sandwich” regimen. In: *American Academy of Dermatology Annual Meeting*. Orlando FL, 2025.

39 J. Tan, J. Q. Del Rosso, J. S. Weiss *et al.* Prevalence and Demographics of Truncal Involvement Among Acne Patients: Survey Data and a Review of the Literature. *J Clin Aesthet Dermatol* 2022; 15: 62-67.

40 Y. R. Woo, H. S. Kim. Truncal Acne: An Overview. *J Clin Med* 2022; 11.

41 J. Y. Ko, C. H. Song, K. J. Kim *et al.* Consensus Report on Truncal Acne: The Korean Acne and Rosacea Society Experts Panel. *Ann Dermatol* 2024; 36: 35-43.
